# Individual differences in task-unrelated thought in university classrooms

**DOI:** 10.3758/s13421-021-01156-3

**Published:** 2021-04-22

**Authors:** Michael J. Kane, Nicholas P. Carruth, John H. Lurquin, Paul J. Silvia, Bridget A. Smeekens, Claudia C. von Bastian, Akira Miyake

**Affiliations:** 1 Department of Psychology, University of North Carolina at Greensboro, P.O. Box 26170, Greensboro, NC 27402-6170 USA; 2 Department of Psychology and Neuroscience, University of Colorado Boulder, 345 UCB, Boulder, CO 80309-0345 USA; 3 Department of Psychology, University of Sheffield, Western Bank, Sheffield, S10 2TN UK

**Keywords:** Mind-wandering, Multitasking, Education, Learning, Interest

## Abstract

This study investigated what academic traits, attitudes, and habits predict individual differences in task-unrelated thought (TUT) during lectures, and whether this TUT propensity mediates associations between academic individual differences and course outcomes (final grade and situational interest evoked by material). Undergraduates (*N* = 851) from ten psychology classes at two US universities responded to thought probes presented during two early-course lectures; they also indicated sitting in the front, middle, or back of the classroom. At each probe, students categorized their thought content, such as indicating on-task thought or TUT. Students also completed online, academic-self-report questionnaires at the beginning of the course and a situational interest questionnaire at the end. Average TUT rate was 24% but individuals’ rates varied widely (*SD* = 18%). TUT rates also increased substantially from the front to back of the classroom, and modestly from the first to second half of class periods. Multiple-group analyses (with ten classroom groups) indicated that: (a) classroom media-multitasking habits, initial interest in the course topic, and everyday propensity for mind-wandering and boredom accounted for unique variance in TUT rate (beyond other predictors); (b) TUT rate accounted for unique (modest) variance in course grades and situational interest; and (c) classroom media multitasking and propensity for mind-wandering and boredom had indirect associations with course grades via TUT rate, and these predictor variables, along with initial interest, had indirect associations with end-of-term situational interest via TUT rate. Some academic traits and behaviors predict course outcomes in part because they predict off-task thought during class.

**Supplementary Information:**

The online version contains supplementary material available at 10.3758/s13421-021-01156-3.

## Introduction

Most research on mind wandering, in which subjects are unpredictably probed to report their immediately preceding thoughts, is conducted in the laboratory to test basic theory about attention and consciousness (e.g., Fox & Christoff, 2018; Smallwood & Schooler, 2015). As research on task-unrelated thought (TUT) has grown, however, so has its study in everyday contexts where distraction may be costly, including aeronautics and astronautics (e.g., Casner & Schooler, 2014; Gontier, 2017), transportation (e.g., Burdett et al., 2019; Walker & Trick, 2018), the workplace (e.g., Dane, 2018; Merlo et al., 2020), and classrooms (e.g., Lindquist & McLean, 2011; Wammes, Boucher, et al., 2016; Wammes, Seli, et al., 2016). In fact, TUTs were first studied empirically in an educational setting (Bloom, 1953).

The present study used the authentic classroom context to ask fundamental questions about individual differences in TUTs and their predictors and consequences: What kinds of students tend to report more TUTs in class, and do those students learn or enjoy the course less? Research that has contrasted daily-life findings with laboratory findings demonstrates that the cognitive-ability, personality, and contextual predictors of TUTs can differ across settings (e.g., Kane et al., 2007; Kane, Gross, et al., 2017; Smeekens & Kane, 2016). Therefore, laboratory studies–like any particular context–provide only partial and circumscribed answers to the field’s theoretical questions. Classrooms are not only an important ecological context for students, but their structure and homogeneity make them strong complements of laboratory contexts in the study of TUTs. Here, we ask whether in-class TUT reports predict academic outcomes beyond the influence of other commonly studied individual-differences variables, and whether classroom TUT rate mediates, in part, the associations between some of these individual-differences predictors and academic outcomes.

### How much, when, and where students mind-wander in class

Bloom’s (1953) seminal study assessed college students’ class-related and class-unrelated thoughts during five lecture and 29 discussion sections across disciplines. Students listened to a recording of a class they attended hours before and were periodically probed for what they were originally thinking in that moment. Bloom characterized students’ thought reports as being task-related or unrelated and found TUT rates of 24% and 12% during lecture and discussion, respectively. The evidence confirmed what every teacher knows from hard experience: Students’ minds frequently wander, even during activities promoting active attention.

Subsequent research in educational settings has assessed TUTs more directly, by probing students’ thoughts in the moment, rather than recalling them later. However, until recently, most classroom studies followed Bloom’s (1953) exclusive focus on estimating TUT prevalence and its contextual variation: Lectures elicit higher TUT rates than do active pedagogical exercises (Acai, 2016; Bunce et al., 2010; Locke & Jensen, 1974; Schoen, 1970), but student-led discussions yield more TUTs than do teacher-led discussions (Cameron & Giuntoli, 1972; Schoen, 1970). Moreover, consistent with Bloom, students zone out not only during lectures, but also during active problem-solving activities (e.g., Geerligs, 1995; Schoen, 1970; Shukor, 2005).

TUT reports also increase with time in class sessions (i.e., more TUTs later than earlier in class) in most studies (Cohen et al., 1956; Lindquist & McLean, 2011; Stuart & Rutherford, 1978; Varao-Sousa & Kingstone, 2019). Although increasing TUT rates fit with laboratory findings (e.g., Kane, Smeekens, et al., 2017; Risko et al., 2012; Wammes & Smilek, 2017), several recent studies have found unchanging or decreasing TUT rates with time in class (Wammes et al., 2019; Wammes, Boucher, et al., 2016; Wammes & Smilek, 2017). More data are needed to address these inconsistencies and explore further whether time-in-class effects distinguish laboratory from classroom TUTs.

Finally, two studies have assessed whether TUTs vary with seating location (Lindquist & McLean, 2011; Wammes et al., 2019), as students sitting closer to the instructor tend to perform better (LaCroix & LaCroix, 2017), perhaps because it facilitates focused attention (Breed & Colaiuta, 1974). One study found more TUTs for students seated further back (Lindquist & McLean, 2011; *N* = 463). The other found no variation in TUTs by seating location, but its restricted range of TUT reports may have limited power to detect any association (Wammes et al., 2019; *N* = 76).

These mixed results regarding time-in-class and seating location call for replication. We examined these issues as a secondary goal of the present study.

### TUT rates and learning in the classroom versus in the lab

Do TUTs have consequences for learning? The correlational nature of mind-wandering research discourages causal claims, but the field has assessed the association between TUT rates during live and online lectures and subsequent learning (e.g., Hollis & Was, 2016; Lindquist & McLean, 2011). Students who report more TUTs during lectures also tend toward poorer comprehension: Most studies find a modest negative correlation (≈−.20) between TUT rates and scores on either same-day quizzes (Wammes, Seli, et al., 2016; Wammes & Smilek, 2017) or later exams (Hollis & Was, 2016; Lindquist & McLean, 2011; Siegel et al., 1963; Wammes, Seli, et al., 2016). Some studies have elicited null associations, however (Varao-Sousa & Kingstone, 2015, 2019; Wammes et al., 2019; Wammes, Seli, et al., 2016).

Although it is not clear why some studies find no TUT–learning correlation, most correlations reported from classroom studies are weaker than those from the lab. When students attempt to learn from recorded lectures in a laboratory setting, TUT rates typically correlate more strongly (*r*s = −.30 to −.50) with lecture comprehension (Jing et al., 2016; Kane, Smeekens, et al., 2017; Loh et al., 2016; Risko et al., 2012, 2013; Varao-Sousa & Kingstone, 2015; but see Was et al., 2019). The weaker TUT–learning correlations in the classroom may be attributable to more variables operating there (e.g., students choosing their courses, attendance rate, study time). Moreover, outcomes assessed in the lab occur temporally close to TUT reports (i.e., immediately post-lecture), so outcomes may be partially reactive to making repeated TUT reports, such as giving up on a test after reporting frequent TUTs. Any such reactivity could artificially drive up the TUT–learning correlation in the lab relative to classroom studies, where outcomes are frequently assessed days or weeks after TUT reports.

Classroom studies are thus critical, as complements to laboratory studies, to estimating the effect size of the TUT–learning association and explore its possible causes. The classroom context can help illuminate important individual-differences variables that predict TUTs and their potential consequences for learning and achievement.

### Exploring individual differences in classroom TUT rate

Most studies that have investigated TUT-learning associations in educational settings have also assessed associations between TUTs and other individual differences. Students’ ratings of their background knowledge in the course do not typically predict TUT rates (Wammes et al., 2019; Wammes, Seli, et al., 2016; Wammes & Smilek, 2017), and only limited evidence suggests that working-memory capacity (WMC; Hollis & Was, 2016), notetaking quantity (Lindquist & McLean, 2011), and seating location (Lindquist & McLean, 2011; but see Wammes et al., 2019) correlate negatively–and modestly–with TUT rate in educational settings. In contrast, the most replicated negative correlates of classroom TUT rate are students’ interest in the course material and their motivation to perform well (in general or in the specific course), with most between −.20 and −.50 (Hollis & Was, 2016; Lindquist & McLean, 2011; Ralph et al., 2017; Varao-Sousa & Kingstone, 2015, 2019; Wammes et al., 2019; Wammes, Seli, et al., 2016; Wammes & Smilek, 2017).

Although these consistent results for interest and motivation are encouraging, their measurement has been rudimentary. First, most studies used one item to assess each construct (e.g., “How interested are you in this topic?”; Hollis & Was, 2016; Lindquist & McLean, 2011; Varao-Sousa & Kingstone, 2015, 2019; Wammes, Seli, et al., 2016; Wammes et al., 2019; Wammes & Smilek, 2017); only the grit construct, which is conceptually related to interest and motivation, has been measured using multiple items (Ralph et al., 2017; Wammes et al., 2019). Second, sometimes interest and motivation have been measured after the lecture or course (Hollis & Was, 2016; Ralph et al., 2017; Varao-Sousa & Kingstone, 2015, 2019); measures taken after a lecture or course cannot be considered *predictors* of TUT rates or learning, and may be reactively contaminated by them (i.e., TUTs may reduce reported interest). Classroom TUT research, then, like all of psychology (Flake et al., 2017; Flake & Fried, 2020), must attend more to measurement.

### TUT rate as a mediating variable

Because several individual-differences variables appear to predict classroom TUT rate, which in turn predicts classroom learning, the propensity for TUTs in class may act as a mediating variable between educationally relevant constructs (e.g., motivation) and outcomes (e.g., exam scores). Perhaps students’ initial topic interest and motivation, for example, are associated with better learning in part because they are associated with less frequent classroom TUTs.

Only two lecture-learning studies (both using videos) have assessed whether TUT-report rate acts as a mediator (Hollis & Was, 2016; Kane, Smeekens, et al., 2017). In an authentic online course (Hollis & Was, 2016), 126 students viewed two of the lectures (13 min each) with four thought probes embedded in each, and then completed a quiz. Before and after each lecture, students rated their interest in the topic on a 1–5 scale and, at some point during the course, students completed three tests of WMC. A structural equation model indicated that both interest (β = −.66) and WMC (β = −.26) factors independently predicted a TUT-rate factor, and TUT rate in turn predicted an outcome factor based on quiz scores and overall course performance (β = −.45); WMC also had a direct association with course outcomes, independent of TUT rate (β = .40). These findings suggest that both interest and WMC had indirect effects on course performance via TUT rate, but no formal mediation tests were reported.^1^

In the laboratory (Kane, Smeekens, et al., 2017), 182 students viewed a 52-min video lecture on statistics with 20 embedded thought probes. Before the video, subjects took a test of WMC and a pretest on statistics (to assess prior knowledge), and completed self-reports on their prior math interest, confidence in learning from the lecture, incremental beliefs in math intelligence, and classroom media multitasking habits (e.g., texting during class).^2^ In a simultaneous regression model, pretest scores (β = −.16), prior math interest (β = −.20), and classroom media multitasking (β = .18) all predicted TUT rate (WMC’s negative association was not significant). In a model predicting post-video test performance, TUT rate had a significant effect (β = −.34) beyond the other predictors; moreover, pretest, prior interest, and classroom multitasking all had significant indirect effects on test performance via TUT rate.

Although limited to video lectures, these studies suggest that knowledge- and interest-based predictors of learning draw some predictive power from their shared variance with TUTs during learning. Moreover, both mediation studies demonstrate that TUT rate predicts learning even when statistically controlling for educationally relevant individual-differences variables that are plausible third-variable candidates. The field needs more such studies to investigate additional, plausible third variables to draw stronger inferences about the potential consequences of classroom TUTs for learning.

### The present study

The present study investigated several academic predictors of classroom TUT reports and assessed educational outcomes that, in turn, might be predicted by propensity for TUTs during class.

#### Methodological strengths and advances

We assessed learning as one critical outcome (operationalized as course grades) and situational interest in the course as another (i.e., topic interest *evoked by* the learning context; Hidi, 1990). Educators strive not only to convey knowledge and habits of thought, but also to motivate students to derive pleasure from, and seek out, learning. Situational interest is therefore an important outcome construct in educational research (e.g., Harackiewicz et al., 2008; Linnenbrink-Garcia et al., 2010). So, as we did in our laboratory study (Kane, Smeekens, et al., 2017), the present study used situational interest and learning as two desirable educational outcomes that may be (negatively) associated with TUT rate.

Kane, Smeekens, et al. (2017) observed that TUT rate predicted both learning from a video lecture on statistics and their reports of how interesting they found the lecture. Moreover, TUT rates predicted this situational interest beyond the influence of students’ *prior interest* (and knowledge) in math. These lab findings suggested a reciprocal relation between TUTs and interest, with low initial interest predicting more TUTs and then more TUTs predicting still decreased situational interest derived from the lecture. The present study sought to evaluate the generalizability of these findings to the classroom context.

The present study also expanded and improved on prior measures of classroom-TUT predictors. First, we assessed all educationally relevant predictor constructs during the second week of class, so they were *predictors* and not reactively affected by classroom experiences of TUT and learning; moreover, TUT rates were measured relatively early in the course, with one assessment in each classroom occurring before the first exam, and so TUT rates (indicating students’ general propensity for off-task thought) may be properly considered *predictors* of course outcomes and minimally contaminated by them.

Second, because studies of classroom TUT have so often used only a single instrument–or a single item–to measure motivation and initial-interest constructs, we measured multiple facets of both motivation (i.e., mastery goal orientation, performance goal orientation, self-efficacy) and prior interest (i.e., topic-interest value, utility value, attainment value, and intrinsic value) that also figure prominently in the literature on individual differences in academic success (e.g., Allen & Robbins, 2010; Pintrich & De Groot, 1990; Robbins et al., 2004, 2006; Schneider & Preckel, 2017).

Third, because prior work included a narrow set of individual-differences variables–and usually only one or two per study–we included several predictors beyond initial interest and motivation. We asked students about their notetaking habits based on prior findings that some aspects of notetaking quality correlate negatively with TUT rate during learning (Kane, Smeekens, et al., 2017; Lindquist & McLean, 2011). We assessed classroom media-multitasking habits because Kane, Smeekens, et al. found that it correlated positively with TUT rate and that TUT rate mediated its association with both learning from, and situational interest in, a video lecture. We measured test anxiety because it not only affects academic performance, but it also is characterized by distracted, preoccupied thinking (e.g., Beilock et al., 2007; Sarason, 1984; Zeidner, 1998). Finally, we measured trait propensity for mind wandering and boredom to test whether our probed, state assessments of TUTs in the classroom predicted academic outcomes beyond a general proneness toward boredom-driven off-task thought.

Finally, classroom studies typically sample TUTs either within a single lecture or within multiple lectures from a single course, thus potentially limiting their findings’ reliability, generalizability, or both. The present study sought greater reliability and generalizability by sampling TUTs within two meetings each from ten different undergraduate classes on two different topics–introductory psychology and psychological statistics–at two different universities, with a sample of 851 students (an unusually large sample for this literature).

#### Study goals

Our primary goals were to: (a) assess the individual-differences predictors of TUT rate, measuring these predictors at the beginning of the course, before our assessments of classroom TUTs; (b) assess the individual-differences predictors of course performance and course situational interest, including TUT rate (measured before the classroom outcome variables were assessed), to test whether propensity for TUTs predicted educational outcomes beyond the potential influences of other academic individual-differences variables; and (c) test for the potential mediating role of TUT rate in the associations between our individual-differences predictor variables and two course outcomes.

Our secondary goals were to follow up on limited prior findings to: (a) assess whether TUT rates increased from the first to second half of class sessions; and (b) test whether sitting in the front, middle, versus back third of the classroom were associated with increasing TUT rates.

## Method

Below we report how we determined our sample size and all data exclusions, manipulations, and measures in the study (Simmons et al., 2012). All questionnaires described below are available at https://osf.io/hptvj/. The study received Institutional Review Board approval from the University of Colorado Boulder (UCB) and the University of North Carolina at Greensboro (UNCG). Both are public universities; UNCG is a minority-serving institution for African-American students. For 2015 freshman cohorts, mean verbal and math SATs at UCB were 606 and 613, respectively, and at UNCG were 520 and 519, respectively.

To preserve student and instructor confidentiality, we hereafter refer to these institutions as University A and University B. In the informational materials and consent document, we assured students that only our research team–not their instructors–would access their data, and that only a list of students who either participated in the study or completed an alternative assignment would be provided to instructors at semester’s end to assign extra credit.

### Subjects

We invited all 1,892 students registered for ten target classes at Universities A and B to participate for extra-credit points (or complete an alternative assignment). These classes represented all seven sections of Introductory Psychology (two at University A, five at University B) and all three sections of Psychological Statistics (two at University A, one at University B) offered during one academic semester; because Introductory Psychology was a prerequisite for Psychological Statistics at both universities, students were not enrolled in both. All ten course instructors were briefed on the plan for the study and agreed to participate. Sample size was determined by participation rates.

Appendix 1 presents the number of students registered for each course, the number who initially consented, and the number who completed all required components. A higher proportion of registered students at University A consented for the study than at University B, but a higher proportion of consented students at University B completed the entire study than at University A.

We consented 851 students (44.9%) who also completed all components for inclusion in data analyses, affirmed use of their data, passed at least three of five attention-check items (see below), and were at least 18 years old. Mean age for students included in analyses was 19.2 years (*SD* = 2.8; *n* = 845 reporting); 75.3% reported their gender as female and 24.7% as male (*n* = 849 reporting). The racial composition (*n* = 840 reporting) was 71.5% White/European American, 13.5% Black/African American, 7.1% Asian American, and 6.9% Multiracial; ethnicity was reported separately (*n* = 849 reporting) and indicated that 9.3% were of Hispanic/Latino(a) heritage.

Appendix 1 also shows subjects’ mean final grades in the course, standardized against all students earning final grades in each class. These *z*-scores indicated some selection bias, with our subjects performing, on average, better than their classmates (all class *M*s > 0), likely because students who fail classes don’t typically attend through semester’s end or complete small extra-credit assignments. Despite the modestly biased sample, the SDs around final grades were substantial, indicating individual differences that might be predicted by our constructs of interest.

### Procedure and materials

The method closely paralleled that from our laboratory study of individual differences in mind-wandering and learning (Kane, Smeekens, et al., 2017), in which students (a) completed questionnaires, (b) viewed a video lecture with thought probes, and (c) took a test of learning and reported situational interest in the lecture. The present study also had three phases, but across a semester. First, students consented and completed a set of trait and behavior questionnaires online, reflecting our academic predictors. Second, students reported on the contents of their immediately preceding thoughts upon auditory experience-sampling probes being presented throughout two early class meetings. Third, at semester’s end, students reported on their situational interest in the course, and the instructors provided us with students’ course grades. These three phases and their materials are detailed below.

#### Phase 1 online questionnaires

During the first week of the 15-week semester, the first author (at UNCG) or last author (at UCB) visited each class to explain the study. During the second week only, students were given access to the consent form and questionnaires via Qualtrics to complete outside of class.

Questionnaires appeared in the order below and took 15–20 min to complete. Table 1 presents sample items for both Phase 1 and Phase 3 instruments. Unless otherwise specified, subjects rated each item on a 1–5 scale labeled “strongly disagree,” “somewhat disagree,” “neither disagree nor agree,” “somewhat agree,” and “strongly agree;” appropriate items were reverse-scored before calculating internal consistencies or averages. Five attention-check items, representing infrequency (e.g., “I write my class notes by alternating between French and Portuguese”) or directed questions (e.g., “To show I am paying attention I will answer ‘usually not true for me’ for this question”), were included to discourage careless responding. We report McDonald’s ω (JASP Team, 2020) for each scale as an internal consistency indicator, as it is psychometrically superior to Cronbach’s α (e.g., McNeish, 2018; Revelle & Zinbarg, 2009; Trizano-Hermosilla & Alvarado, 2016).

**Table 1:** Sample items from questionnaires

Phase and questionnaire	Example items *[item type, where applicable]*
*Phase 1, Beginning-of-Semester*	
Note-taking skill	It is hard for me to take notes in class, keep up with the instructor, and understand the concepts at the same time.
	I can take notes on material that is boring, technical, or overly complicated.
Multitasking beliefs	My ability to learn in class while multitasking is: _______ *[engagement]*
	Multitasking in class is perfectly fine as long it doesn’t hurt my grades. *[beliefs]*
Interest and value	I enjoy learning [topic]. *[interest value]*
	It is important for me to be a person who reasons [topically]. *[attainment value]*
	[Topic] is practical for me to know. *[utility value]*
	I prefer class work that is challenging so I can learn new things. *[intrinsic value]*
Mastery achievement goals	I am striving to understand the content of this course as thoroughly as possible. *[approach]*
	My aim is to avoid learning less than I possibly could. *[avoidance]*
Performance achievement goals	My goal is to perform better than the other students. *[approach]*
	My goal is to avoid performing poorly compared to others. *[avoidance]*
Course self-efficacy	I’m certain I can understand the ideas taught in this course.
	I think I will receive a good grade in this class.
Test anxiety	I worry a great deal about tests.
	When I take a test, I think about how poorly I am doing.
Mind-wandering & boredom	At times it is hard for me to keep my mind from wandering. *[mind-wandering]*
	I find that I easily lose interest in things that I have to do. *[boredom]*
*Phase 3, End-of-Semester*	
Situational interest	I enjoyed coming to the lecture. *[interest in course]*
	I found the content of this course personally meaningful. *[utility/value of course]*
	[Topic] fascinates me. *[interest/value in discipline]*

*Note-taking skill*. This 11-item scale (Kane, Smeekens, et al., 2017) asked about note-taking habits and skills. Subjects responded to each item using a 1–5 scale, labeled “Never,” “Rarely,” “Sometimes,” “Often,” and “Always,” respectively. We averaged the last eight items only, as the first three asked about note-taking method (e.g., on paper or via computer) rather than about skill (ω = .68).

*Classroom media multitasking.* From a seven-item scale that assessed a variety of behaviors in classrooms (e.g., doodling, talking, daydreaming), we followed Kane, Smeekens, et al. (2017) and averaged only the first three items that asked about engaging with electronic media during class. Subjects reported, using the same 1–5 scale as in the note-taking questionnaire, how frequently they engaged in “texting, IM’ing/chatting, or tweeting,” “checking and sending emails,” and “web surfing (including social media sites),” during class (ω = .83).

*Classroom multitasking beliefs.* In a measure adapted from Sanbonmatsu et al. (2013), subjects completed six items asking about engagement and success in daily-life and classroom multitasking, responding via a 1–5 scale labeled “much less than average,” “somewhat less than average,” “about average,” “somewhat more than average,” and “much more than average,” and five items on a 1–5 agreement scale asking about their beliefs about the harm of multitasking in class. We first created two subscales of three items each for items about engagement (ω = .49) and success (ω = .54) and a subscale for five items about harm (ω = .69), and then created an overall score by averaging the three subscales (ω = .70).

*Topic interest and value.* Subjects completed 24 items assessing their initial interest in the course topic and its motivational value. Items were derived from measures of interest value (five items; ω = .91), attainment value (five items; ω = .90), utility value (five items; ω = .87), and intrinsic value (nine items; ω = .80; Conley, 2012; Linnenbrink-Garcia et al., 2010; Pintrich & De Groot, 1990; Pintrich et al., 1991); the intrinsic value items were presented to subjects later, intermixed with the self-efficacy and text anxiety items (described below). For students in introductory psychology, the course topic was labeled “psychology,” and for students in statistics, it was labeled “mathematics/statistics.” We created a subscale for each of the value types and then averaged the subscales into an overall score (ω = .88).

*Course self-efficacy.* We assessed self-efficacy for learning and performance for the target course with a nine-item scale from Pintrich and De Groot (1990; ω = .86). Items were presented amid intrinsic value items (described above) and test anxiety items (described below).

*Test anxiety.* Four items from Pintrich and De Groot (1990) asked students about test anxiety (ω = .89). Items were presented amid intrinsic value and self-efficacy items (described above).

*Achievement goals (mastery and performance).* Six items (from Elliot & Murayama, 2008) assessed approach or avoidance mastery goals (to learn material), and six assessed approach or avoidance performance goals (to perform well); mastery orientations generally predict more intrinsic motivation and better long-term learning and achievement than do performance orientations (e.g., Elliot & Harackiewicz, 1994; Elliot & Church, 1997). We created subscales for mastery approach (ω = .76), mastery avoidance (ω = .68), performance approach (ω = .84), and performance avoidance items (ω = .80); we averaged mastery subscales (*r* = .28) into a mastery goals score and performance subscales (*r* = .55) into a performance goals score.

*Mind-wandering and boredom proneness.* Two scales of the Imaginal Process Inventory (Singer & Antrobus, 1970) assessed proclivities for distracted mind-wandering (12 items) and boredom (12 items). Each item provided a 1–5 response scale, labeled “definitely not true for me,” “usually not true for me,” “usually true for me,” “true for me,” and “very true for me,” respectively. We created a subscale for mind-wandering (ω = .84) and boredom (ω = .76) and averaged them together (*r* = .52).

#### Phase 2 classroom thought reports

**Fig. 1:**
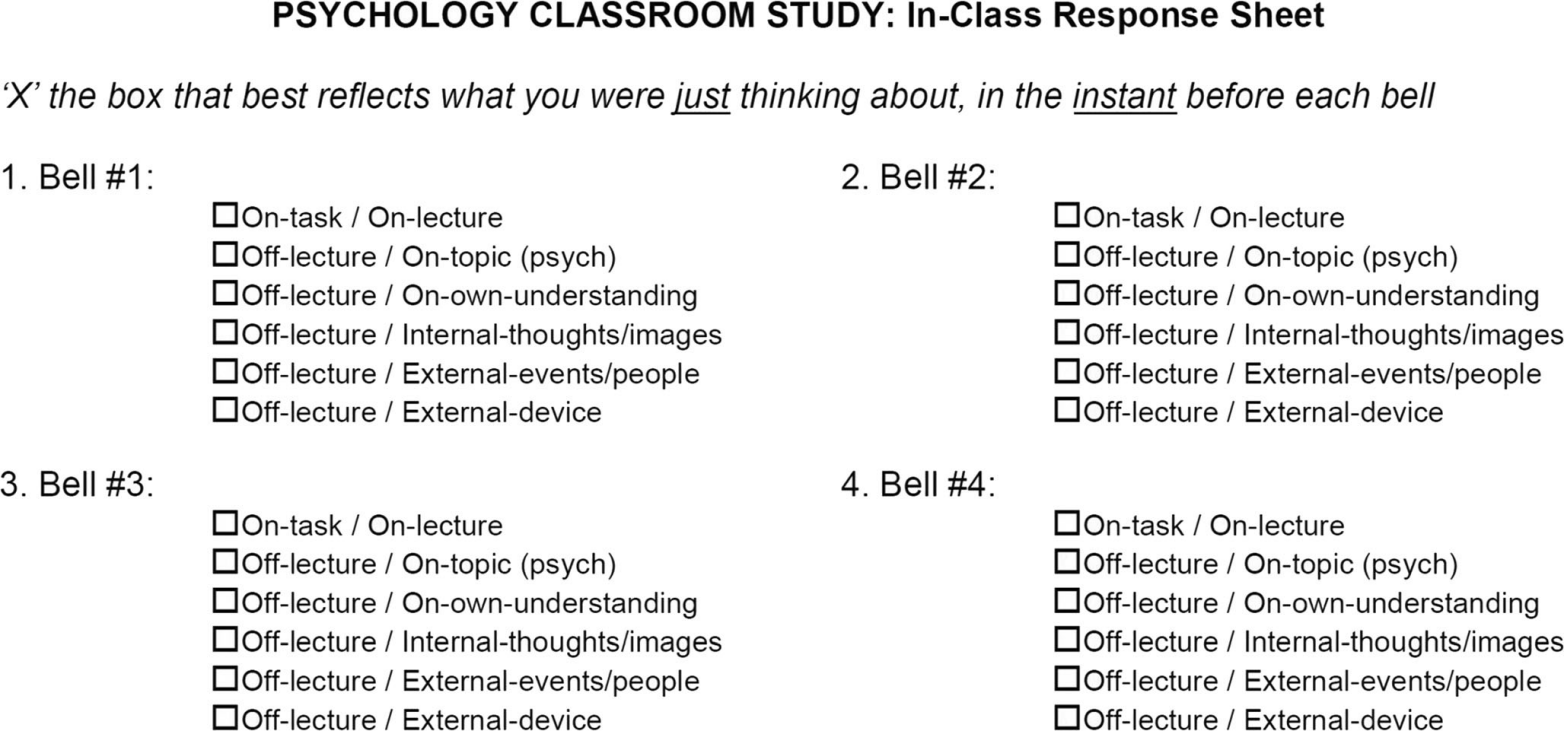
Top portion of the in-class thought probe response sheet

We assessed students’ in-the-moment thought content during two sessions of each class. For all classes, the first classroom visit was 1–2 weeks after Phase 1 and before the first exam (4–5 weeks into the course); the second visit was 1–2 weeks after the first exam. On the day before each classroom visit, instructors emailed students a reminder to attend. We retained and analyzed data from students who provided thought reports from at least one of the two visits (*n* = 732 with reports from two visits, *n* = 59 with reports from only visit 1, and *n* = 60 with reports from only visit 2). At the start of each visit, the first author (at UNCG) or last author (at UCB) reminded students about the study and explained the thought-probe signals and response sheets (see Fig. 1).

Each thought probe was signaled by an experimenter in the very back of the lecture hall (in most classrooms, situated on a platform behind the last row of seats), ringing a Schwinn Classic bicycle bell (model SW77724-6); between probes the experimenter was silent. Probes occurred as close as possible to a prespecified list of times, nine for 75-min classes and six for 50-min classes.^3^ All classes followed one list of randomized probe times for the first class meeting (at 11, 15, 20, 26, 28, 38, 48, 62, and 65 min) and another list of times for the second meeting (at 9, 13, 23, 27, 33, 37, 46, 53, and 61 min). Each list was randomized with the constraints that no probes could appear during the first or last 5 min of the class, and that three probes would appear within each remaining eligible 20 min segment of the course. At each bell, a second experimenter seated toward the front of the room held up a sign with the number of that probe to help students use the correct space on their probe response sheet.

We note, however, that probes did not always occur at these prespecified times because we assured instructors that we would not ring the bell if they or a student were speaking. If the instructor or a student was speaking at the prespecified probe time, then the experimenter waited to ring the bell until they judged the speaker to have finished. In most instances, probes occurred at a moment when the instructor had been talking, but probes sometimes occurred following a student question, during a video presentation, or during a discussion exercise. We broadly noted the course activity at each probe but did not formally code them or analyze associations between concurrent activities and TUT rates.

All students were offered a probe response sheet, allowing non-participants to be non-identifiable to instructors. The front side of the sheet (see Fig. 1) instructed students to choose, for each probe, the description that most closely matched “what [they] were *just* thinking about, in the *instant* before each bell.” It then listed 12 bells (Bell #1 to Bell #12), even though students only heard six or nine bells, to obscure when the last one would be.

Under each bell number were six thought-content options, with an empty box next to each. We instructed subjects to check the *one* box that best reflected what they were thinking before that bell. These choices were explained to students as follows (the italicized labels appeared on response sheets):

*On-task/On-lecture*: For thoughts about the course material that was being taught or discussed at the moment.

*Off-lecture/On-topic*: For thoughts about the course topic (e.g., statistics), that did not reflect the here-and-now of the class discussion, such as material from earlier in the lecture or the course, or a real-life example of the topic generated by the student.

*Off-lecture/On-own-understanding*: For thoughts about how well or poorly one is understanding the lecture or discussion.

*Off-lecture/Internal-thoughts/images*: For thoughts unrelated to the lecture or discussion and unrelated to the current surroundings, such as mind-wandering about personal concerns or daydreaming about fantastical scenarios.

*Off-lecture/External-events/people*: For thoughts about objects or events in the current classroom environment unrelated to the lecture or discussion.

*Off-lecture/External-device*: For thoughts about what they had recently seen or read on their laptop, tablet, or phone, that were unrelated to the lecture or discussion.

For all analyses, we used the proportion of probes on which subjects endorsed the last three options (i.e., *internal-thoughts/images*, *external-events/people*, or *external-device*) to indicate TUT rate. Any ambiguous or blank probe responses were scored as missing data; of 851 subjects, 15 had one missing observation, two had two missing, and one had three missing.

On the back of each response sheet were nine questions that students completed at the end of each classroom visit (classes ended 5 min early to facilitate completion). All but Question 8 were included for exploratory, pilot purposes and asked about students’ experiences in that class session. Question 8 asked students to indicate whether they were sitting in the front third, middle third, or back third of the classroom. We report analyses for these data.

#### Phase 3 online questionnaires and course grades

During the last week of class, students completed additional online questionnaires via Qualtrics. Only one was an outcome of primary concern: students’ situational interest in the course and topic (following Kane, Smeekens, et al., 2017). The remaining post-course questionnaires, included for pilot purposes, were not analyzed here as they do not serve as either predictor or outcome variables.^4^

The situational interest survey (see Table 1), adapted from Linnenbrink-Garcia et al. (2010), asked three types of questions about the course (with “psychology” or “statistics” wording used): (a) seven items about how interesting they found the class and the instructor; (b) five items about how useful and valuable they found the course; and (c) five items about how interesting and valuable they found the course discipline. We averaged items for each of the three subscales separately (ωs = .93, .89, and .93, respectively), and then averaged those three scores into a situational interest score (ω = .90).

At semester’s end, instructors provided final numerical course grades. For both introductory psychology and statistics courses, final grades were determined primarily (if not completely) by in-class exams, but statistics courses included more weight on other assignments. We *z*-scored final grades within class sections as our performance outcome.

## Results

Anonymized aggregated data are available at https://osf.io/hptvj/ to allow reproduction of analyses (course grades are z-scored for confidentiality). We adopted α = .05 throughout. Before assessing the mediating role of TUTs, we first consider the key descriptive findings.

### Preliminary analyses: Descriptive statistics

Appendix 2 presents mean rate of TUT reports in each classroom, averaged across both classroom visits, with TUT rates expressed as a proportion of all thought reports (Supplemental Table S1 separately presents visits 1 and 2; see Online Supplementary Material, OSM). TUTs were reported as a common classroom experience, but more common for some students than others. Combined over class visits, mean TUT rates ranged from .17–.31 across classrooms (with *SD*s of .14–.22). Collapsed across all classrooms and visits, students reported TUTs at a mean rate of .24 (*SD* = .18). Students thus reported not attending to class lecture and discussion about a quarter of the time, with TUT rates of about .05–.45 being within 1 *SD* of the mean.

TUT individual differences were reliable, despite well-established state and contextual influences (e.g., Antrobus et al., 1966; McVay & Kane, 2013; Robison et al., 2021; Smallwood et al., 2009). For the 732 students who attended both classroom visits, TUT rates during visit 1 and visit 2 correlated at *r*(730) = .48, 95% CI [.42, .53]. Students who reported more TUT experiences during one class also tended to report more TUTs during another class, several weeks later.

**Fig. 2:**
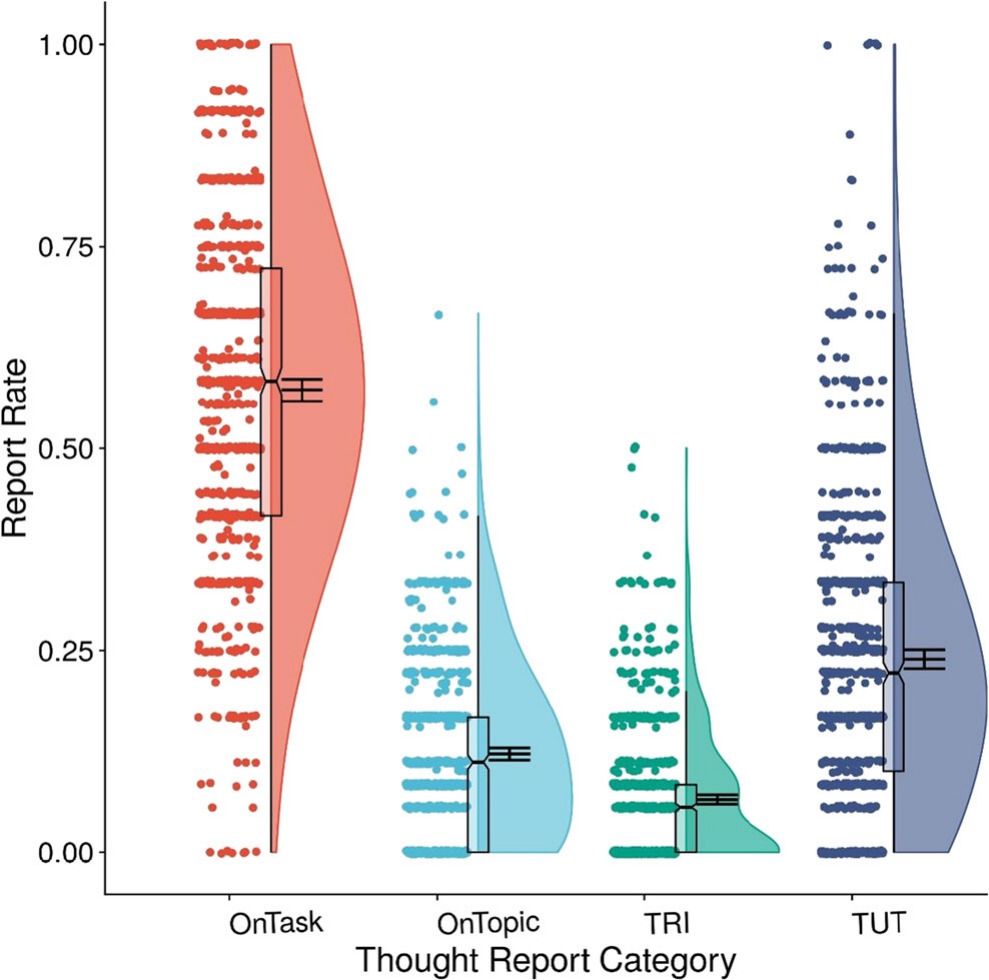
Subjects’ rates for each thought report category as a proportion of all thought reports. OnTask = on-task thoughts about the here-and-now of the lecture; OnTopic = thoughts not about the here-and-now but a class-relevant topic; TRI = “task-related interference,” or thoughts about one’s own understanding of the material; TUT = task-unrelated thought. Boxplots present the 25th, 50th, and 75th percentiles; whiskers extend to the smallest and largest values within 1.5 times the interquartile range. Means and 95% confidence intervals are presented to the right of boxplots; circles represent individual subjects’ thought-report rates

For completeness, Fig. 2 presents raincloud plots (Allen et al., 2019) of rates for the four major thought-report categories, including TUTs, averaged across class visits. Rates of topic-related off-task thought reports (response option 2; “OnTopic”) and comprehension-related off-task thought reports (option 3; “task-related interference” [TRI]) were low and unreliable: Their between-visit correlations were *r*(730) = .24 [.17, .31] and *r*(730) = .16 [.09, .23], respectively. Given their low rates and poor reliabilities, we do not analyze them further.

Finally, Table 2 presents descriptive statistics for our academic predictor variables (phase 1 questionnaires). All had reasonable mean, skewness, and kurtosis values.

**Table 2:** Descriptive statistics for predictor variables from Phase 1 online survey

Measure	M	SD	Min	Max	Skewness (SE)	Kurtosis (SE)
Note-taking skill	3.50	0.48	2.00	5.00	-0.31 (0.08)	0.19 (0.17)
Classroom media multitasking	2.12	0.84	1.00	5.00	0.61 (0.08)	-0.02 (0.17)
Multitasking beliefs	2.89	0.45	1.51	4.73	0.24 (0.08)	0.38 (0.17)
Topic interest and value	3.80	0.69	1.17	5.00	-0.66 (0.08)	0.35 (0.17)
Mastery achievement goals	3.91	0.64	2.00	5.00	-0.14 (0.08)	-0.46 (0.17)
Performance achievement goals	4.08	0.75	1.00	5.00	-0.94 (0.08)	1.38 (0.17)
Course self-efficacy	3.87	0.53	1.25	5.00	-0.44 (0.08)	0.99 (0.17)
Test anxiety	3.36	1.07	1.00	5.00	-0.42 (0.08)	-0.69 (0.17)
Mind-wandering and boredom	3.08	0.46	1.67	4.58	0.08 (0.08)	0.18 (0.17)

Data collapsed across sites and course sections; total N = 851

### Preliminary analyses: Within-class timecourse of TUTs

To allow multiple observations per time-period per subject (and thus reasonably stable estimates), we calculated a TUT rate for each subject from the first half and second half of each lecture’s probes; for sessions with odd numbers of probes, we eliminated the middle probe. For students with data from both classroom visits, we averaged the first- and second-half TUT rates across visits; for students with data from only one visit, we used data from this single visit.

Average TUT rates increased modestly but significantly from the first half (*M* = .213, *SD* = .212) to the second half (*M* = .265, *SD* = .232) of lectures, *t*(850) = 6.26, *p *< .001, *d* = .214 [95% CI: .146, .282]. This timecourse effect remained significant in a repeated measures ANCOVA with class section as a covariate, *F*(1,849) = 7.59, *p* = .006, η_p_^2^ = .009 (section showed no significant effects). It thus appears that students experienced more off-task thoughts as class proceeded.

**Fig. 3:**
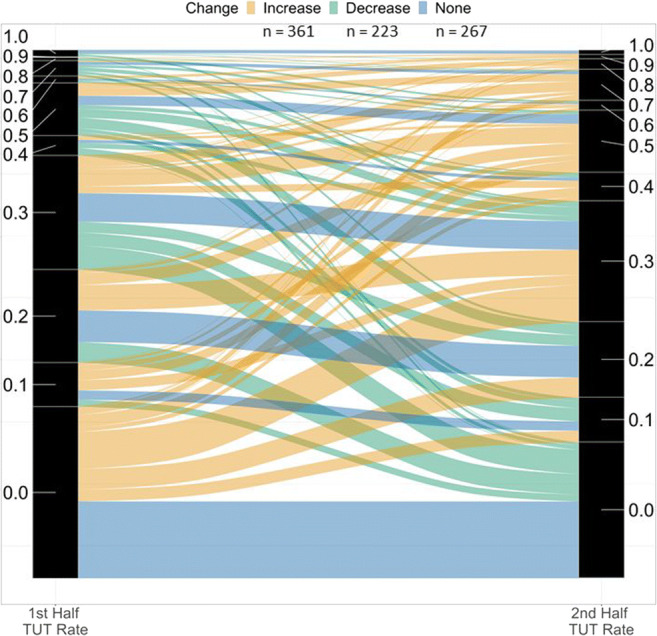
Subjects’ changes in task-unrelated thought (TUT) rate from the first to the second half of in-class probes. Ribbon width reflects number of subjects. Yellow ribbons show subjects whose TUT rate increased from the first to the second half (*n* = 361), green ribbons show subjects whose TUT rate decreased (*n* = 223), and blue ribbons show subjects whose TUT rate did not change (*n* = 267); some blue ribbons slope slightly downward, artifactually, due to changes in TUT-rate bin sizes from first half to second half)

However, upon closer inspection we found that mean TUT rates increased significantly despite more subjects showing either no numerical change (*n* = 267) or a numerical decrease (*n* = 223) in TUT rates across halves than subjects showing a numerical increase (*n* = 361). To visualize these trajectories for 851 subjects, we rounded each subject’s TUT rate to the nearest 0.1 and plotted their first- to second-half changes in the alluvial plot in Fig. 3 (Brunson, 2020); ribbon widths reflect the number of subjects with each trajectory. Subjects showing TUT increases are represented by gold ribbons, subjects showing no change by blue ribbons, and showing decreases by green ribbons (some blue ribbons, for subjects showing no change, artifactually slope slightly downward due to TUT-rate bin sizes changing from first- to second-halves). As the plot indicates, TUT-rate trajectories were not uniform across subjects, which explains the small effect size here and perhaps also the pattern of mixed evidence in the literature.

To explore whether these individual differences in TUT-rate trajectories were systematic, we correlated a change difference score (second- minus first-half TUT rate) with our outcome and predictor variables. TUT-rate change correlated weakly (but just significantly) with final course grade, *r*(849) = −.089 [−.155, −.021], *p* = .010, and post-course situational interest, *r*(849) = −.068 [−.135, −.001], *p* = .048. Students whose TUT rates increased more within sessions earned lower final course grades and developed less situational interest. These correlations are weak enough, however, to warrant skepticism until they are replicated. None of the academic predictor variables correlated significantly with TUT-rate change (all absolute-value *r*s = .005–.066, all *p*s = .892–.055).

### Preliminary analyses: Seating location

**Fig. 4:**
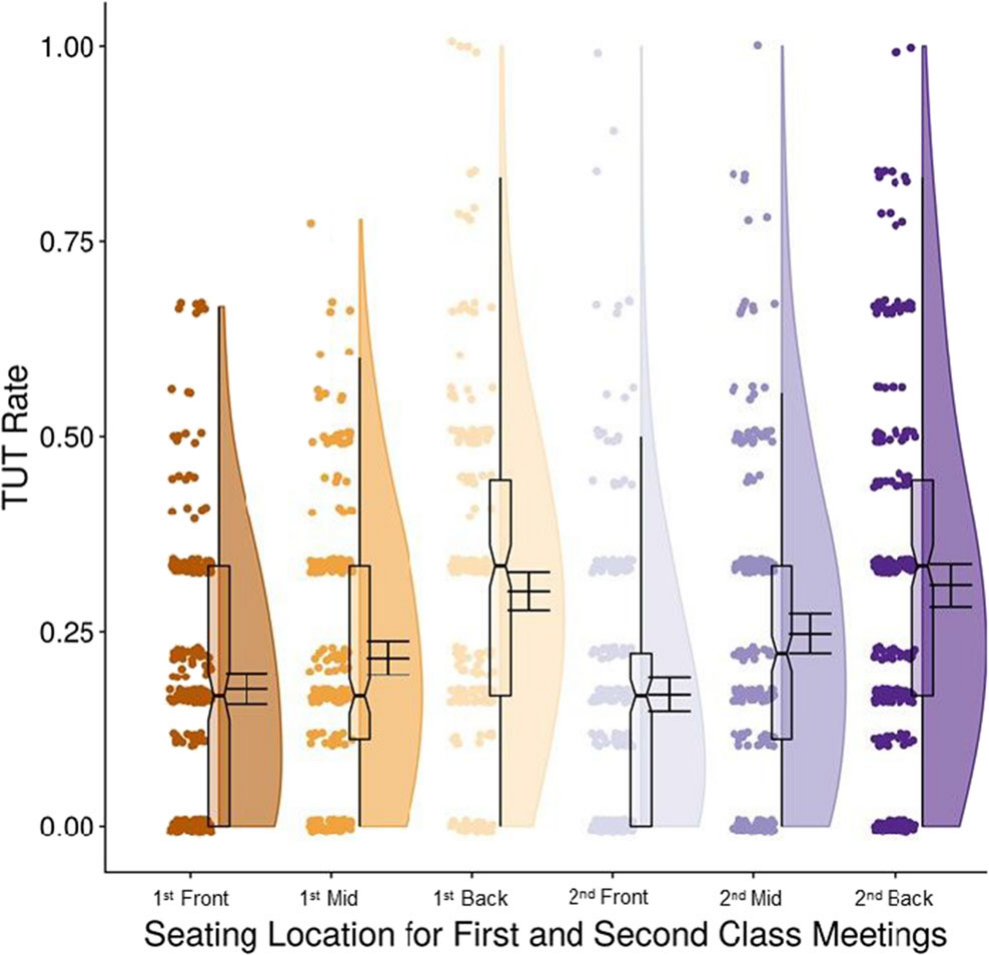
Task-unrelated thought (TUT) rates for the first and second thought-probed class meetings for students seated in the front (“Front”), middle (“Mid”), and back third (“Back”) of classroom rows. Boxplots present the 25th, 50th, and 75th percentiles; whiskers extend to the smallest and largest values within 1.5 times the interquartile range. Means and 95% confidence intervals are presented to the right of boxplots; circles represent individual subjects’ TUT rates

Analyses of seating location (front, middle, back third of classrooms) were correlational because students selected their seats. We analyzed each classroom visit separately because students could change seating locations across classes (of the 726 students with seating data for both visits, 188 changed locations). Figure 4 presents TUT rates for each seating group for each class visit, collapsed over classrooms: TUT rates were markedly higher for students sitting toward the back of the classroom, increasing by 67% and 82% between the front and back third, for the first and second visits, respectively.

For TUT rate during the first visit, ANOVA indicated a significant increase in TUT reports with seating distance, *F*(2,787) = 32.41, *p *<.001, ω_p_^2^ = .074; Tukey post hoc tests indicated that TUT rates increased significantly from students seated in the front (*M* = .18; *n* = 247) versus middle third (*M* = .22; *n* = 248), *t* = 2.30, *p* = .033, and from the middle to the back third (*M* = .30; *n* = 268), *t* = 5.22, *p *<.001. During the second visit, TUT reports similarly increased with seating distance, *F*(2,783) = 31.92, *p *<.001, ω_p_^2^ = .073; post hoc tests again indicated that TUT rates increased significantly from the front (*M* = .17; *n* = 271) to the middle third (*M* = .25; *n* = 240), *t* = 4.33, *p *<.001, and from the middle to the back third (*M* = .31; *n* = 275), *t* = 3.38, *p* = .002.

We followed up these TUT–seating findings with ANCOVAs, first to account for effects of classroom and, second, to additionally account for academic traits and habits that might affect seating choices and thus artifactually drive the seating–TUT association. The effect of seating on TUTs remained significant with classroom as a covariate: for visit 1, *F*(2,786) = 32.38, *p *<.001, η_p_^2^ = .076; for visit 2, *F*(2,782) = 31.88, *p *<.001, η_p_^2^ = .075 (classroom had no measurable effect on TUT rate, either at classroom visit 1, *F*[2,786] = 1.02, *p* = .31, or visit 2, *F*[2,782]<1, *p* = .95). 5 The second ANCOVA additionally included all Phase-1 predictor measures, along with classroom, as covariates. Again, the seating effect on TUTs remained significant: for visit 1, *F*(2,777) = 18.36, *p *<.001, η_p_^2^ = .045; for visit 2, *F*(2,773) = 22.44, *p *<.001, η_p_^2^ = .055. At least for the constructs we measured, then, the effect of seating on TUTs was not driven by academic attitudes or behaviors, or their influence on seating. In fact, classroom media multitasking habits was the *only* predictor in the model that varied with seating location when tested individually for both class visits (multitasking beliefs varied significantly in only classroom visit 2). See Supplementary Table S2 (OSM) for means and ANOVA results for the predictor variables by seating.

### Primary analyses: Correlations

Table 3 presents academic predictor (from Phase 1) correlations with TUT rate (from Phase 2), course grades (from Phase 3), and evoked situational interest (from Phase 3). These correlations are based on the full sample and do not reflect the nested structure of the data (i.e., students within classrooms), as our subsequent multiple-group analyses will.

**Table 3:** Correlation matrix for predictor and outcome variables

	1.	2.	3.	4.	5.	6.	7.	8.	9.	10.	11.	
1. TUT rate												
2. Final course grade z-score	−.14											
3. Situational interest	−.23	.23										
4. Note-taking skill	−.14	.13	.18									
5. Classroom media multitasking	.34	−.17	−.17	−.13								
6. Multitasking beliefs	.06	−.07	−.01	.25	.11							
7. Topic interest and value	−.12	.02	.61	.19	−.18	−.00						
8. Mastery achievement goals	−.11	.07	.27	.15	−.15	−.00	.34					
9. Performance achievement goals	.01	−.00	−.00	.05	.03	.03	.02	.35				
10. Course self-efficacy	−.07	.11	.25	.44	−.06	.13	.39	.24	.18			
11. Test anxiety	.06	−.20	−.09	−.33	.10	−.15	−.04	.02	.11	−.35		
12. Mind-wandering & boredom	.21	−.05	−.13	−.39	.19	−.18	−.14	−.15	−.02	−.35	.28	

Data collapsed across sites and course sections (total *N* = 851). *TUT* task-unrelated thought. Correlations ≥ .07 and ≥ .10 are significant at *p* < .05 and *p* < .005, respectively

Most correlations were modest, but in-class TUT rate correlated significantly with course grade, *r*(849) = −.14 [−.21,−.07], and end-of-semester situational interest, *r*(849) = −.23 [−.29,−.17]. TUT rate, in turn, correlated significantly with most Phase 1 predictor variables, but most strongly (*r*>.20) with classroom media-multitasking habits, *r*(849) = .34 [.28,.40] and everyday proneness for mind-wandering and boredom, *r*(849) = .21 [.14,.27]. Beyond TUT rate, the strongest correlate of course grades was test anxiety, *r*(849) = −.20 [−.26,−.13], and the strongest correlates of situational interest were initial topic interest and value, *r*(849) = .61 [.57,.65], mastery achievement goals, *r*(849) = .27 [.21,.33], and self-efficacy, *r*(849) = .25 [.19,.31].

We dropped two predictor variables from subsequent analyses that failed to correlate at *r* **≥**.10 (*p *<.005) with either TUT rate, course grade, or situational interest: classroom multitasking beliefs (*r*s = −.07 to −.01), and performance goals (*r*s = −.00 to .01).

### Primary analyses: Multiple-group analyses of direct and indirect effects

Our regression-based analyses assessed which individual-differences variables accounted for significant variance in our mediator (TUT rate) or outcomes (course grades and situational interest) beyond that accounted for by other predictors. In the models below, direct effects refer to associations between predictors and outcomes that were not mediated by TUT rate, whereas indirect effects refer to associations between predictors and outcomes mediated by TUT rate.

Nested data, with students grouped into classrooms, are ideally analyzed with multilevel models. However, these methods are not recommended for datasets with fewer than 30 clusters (e.g., McNeish & Stapleton, 2016a), and so our ten classrooms preclude multilevel modeling. An effective–and perhaps ideal–way to model multilevel data with few clusters is with fixed-effects models (McNeish & Kelly, 2019), which can be specified by creating predictors that dummy-code cluster membership or by specifying each cluster as a group in a multiple-group structural equation model that constrains the paths and variances to be equal across groups (McNeish & Stapleton, 2016b, p. 511). These are equivalent models that yield identical estimates, so we selected the multiple-group specification because it is more convenient for path models with indirect effects.

We conducted the analysis in Mplus 7.31 (Muthén & Muthén, 2012), in which the classrooms were specified as groups and the regression coefficients and variances were constrained to be equal across groups (McNeish & Stapleton, 2016b). The models estimated direct effects and indirect effects mediated by TUT rate.

*Direct effects.* Table 4 presents the estimated direct effects (unstandardized) of the predictor variables on classroom TUT rate, final course grades, and post-course situational interest, from the multiple-group analyses. Models tested for the outcomes of course grades and situational interest, with both including TUT rate as a mediator. Significant unique variance in classroom TUT rate was predicted by propensity for classroom media multitasking (more multitasking, higher TUT rate), initial topic interest and value (more prior interest and value, lower TUT rate), and proneness toward mind-wandering and boredom (more mind-wandering and boredom, higher TUT rate).

**Table 4:** Multiple group analysis results for direct effects of each predictor variable on the mediator and the outcome variables

Predictor Variables	On Mediator Variable			On Outcome Variables					
	Classroom TUT rate			Final Course Grade			Situational Interest		
	B	SE	*p*	B	SE	*p*	B	SE	*p*
Note-taking skill	−.016	.015	.260	.124	.069	.071	.110	.061	.072
Classroom media multitasking	**.064**	**.008**	**<.001**	**−.115**	**.035**	**.001**	−.013	.028	.633
Topic interest and value	**−.028**	**.011**	**.012**	−.069	.046	.136	**.540**	**.044**	**<.001**
Mastery achievement goals	−.006	.009	.531	.068	.047	.145	**.131**	**.039**	**.001**
Course self-efficacy	.011	.013	.400	.082	.061	.176	−.059	.054	.278
Test anxiety	−.003	.006	.666	**−.129**	**.028**	**<.001**	**−.059**	**.023**	**.011**
Mind-wandering and boredom	**.059**	**.015**	**<.001**	.108	.065	.096	.053	.057	.348
Classroom TUT Rate				**−.405**	**.164**	**.014**	**−.809**	**.141**	**<.001**

Groups correspond to the ten sampled classrooms (total N = 851). Statistically significant coefficients are presented in bolded type

*TUT* task-unrelated thought, *B* unstandardized coefficient estimate, *SE* standard error

TUT rate, in turn, accounted for significant unique variance in course grades (higher TUT rate, lower grades), as did propensity for classroom media multitasking (more multitasking, lower grades) and test anxiety (more anxiety, lower grades). TUT rate also accounted for significant unique variance in post-course situational interest (higher TUT rate, lower situational interest), as did initial topic interest and value (more initial interest and value, higher situational interest), mastery achievement goals (more mastery orientation, higher situational interest), and test anxiety (more anxiety, lower situational interest). As in our laboratory study (Kane, Smeekens, et al., 2017), then, TUT rate predicted learning and interest outcomes beyond the statistical effects of several academic traits and behaviors.

*Indirect effects.* Unstandardized estimates for indirect effects of our predictor variables on our outcome variables, mediated by TUT rate, are presented in Table 5. For final course grade, both classroom media multitasking and proneness for mind-wandering and boredom had significant indirect effects mediated by classroom TUTs (despite mind-wandering and boredom proneness having no direct effect on grades). For situational interest, significant indirect effects were found again for classroom media multitasking and proneness for mind-wandering and boredom (with neither having direct effects on situational interest), but also for initial topic interest and value.

**Table 5:** Multiple group analysis results for indirect effects of each predictor variable on the two outcome variables via TUT rate

Predictor Variables	Outcome Variables					
	Final Course Grade			Situational Interest		
	B	SE	*p*	B	SE	*p*
Note-Taking Skill	.007	.006	.299	.013	.012	.272
Classroom Media Multitasking	**−.026**	**.011**	**.015**	**−.051**	**.010**	**<.001**
Topic Interest and Value	.011	.007	.085	**.023**	**.010**	**.019**
Achievement Goals, Mastery	.002	.004	.554	.005	.007	.535
Course Self-Efficacy	−.004	.006	.429	−.009	.011	.405
Test Anxiety	.001	.003	.673	.002	.005	.668
Mind-Wandering and Boredom	**−.024**	**.012**	**.045**	**−.048**	**.014**	**.001**

Groups correspond to the ten sampled classrooms (total N = 851). Statistically significant coefficients are presented in bolded type

*TUT* task-unrelated thought, *B* unstandardized coefficient estimate, *SE* standard error

**Fig. 5:**
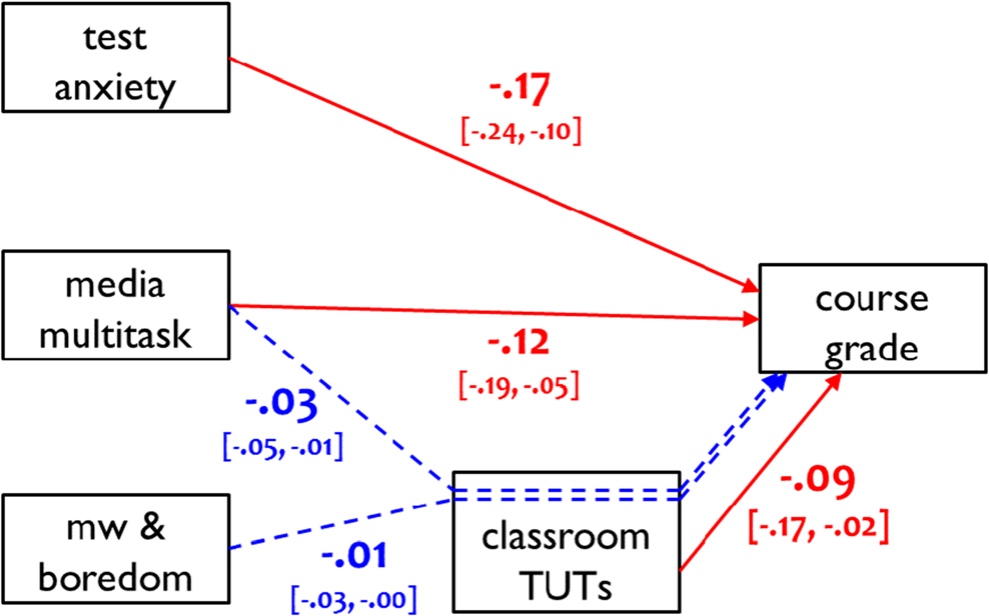
Standardized coefficients for direct and indirect effects of the statistically significant predictors of final course grade, with classroom mind-wandering (TUTs) rate as the mediator variable (bracketed text indicates 95% confidence intervals). Red arrows and coefficients indicate negative direct effects and blue arrows and coefficients indicate indirect effects. “Media multitask” = classroom media multitasking; “MW & boredom” = mind-wandering and boredom; TUTs = task-unrelated thoughts

**Fig. 6:**
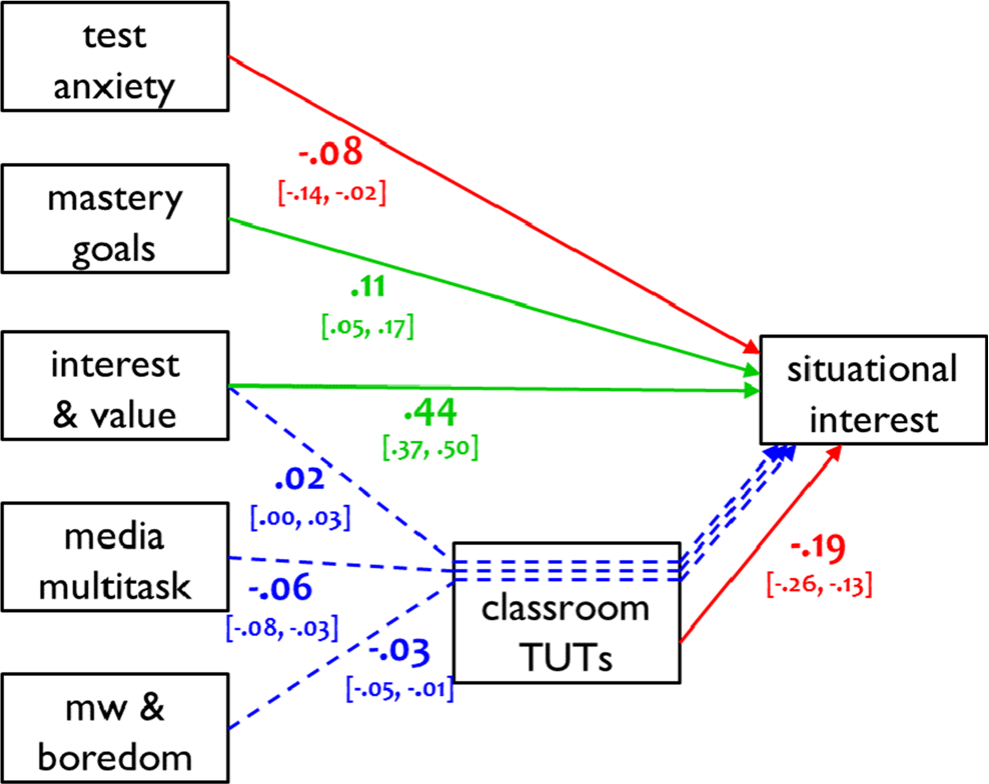
Standardized coefficients for direct and indirect effects of the statistically significant predictors of post-course situational interest, with classroom mind-wandering (TUTs) rate as the mediator variable (bracketed text indicates 95% confidence intervals). Green arrows and coefficients indicate positive direct effects, red arrows and coefficients indicate negative direct effects, and blue arrows and coefficients indicate indirect effects. “Interest & value” = topic Interest and value; “Media multitask” = classroom media multitasking; “MW & boredom” = mind-wandering and boredom; TUTs = task-unrelated thoughts

To visualize all significant predictor pathways, Figs. 5 and 6 present standardized estimates of the direct and indirect effects on final course grade and situational interest, respectively. All indirect effects mediated by TUT rate are indicated by dotted blue lines. Unmediated direct effects are indicated by green and red solid lines, for positive and negative associations, respectively.

*Analyses restricted to Introductory Psychology.* Because we sampled from two course types, Introductory Psychology and Statistics, some of the reported effects may have been driven by one domain. Indeed, test anxiety might plausibly predict outcomes more strongly in statistics than psychology courses, given the high prevalence of math anxiety and its strong association with test anxiety (Ashcraft & Ridley, 2005). We therefore reconducted the multiple-group analyses for only the introductory psychology classes, which had enough sections and students to analyze with confidence (seven sections; *n* = 654). Supplemental Tables S3 and S4 (OSM) present statistics for direct and indirect effects, respectively.

As in the full sample, TUT rate was significantly predicted by classroom media-multitasking habits (positively), mind-wandering and boredom proneness (positively), and initial topic interest and value (negatively). For course grades, direct effects were again found for TUT rate (negative), classroom media multitasking (negative), and test anxiety (negative), but here, additionally, for mastery goals (positive); a significant indirect effect mediated by TUT rate was again found for classroom media multitasking, but here the indirect effect for mind-wandering and boredom proneness was not significant (*p* = .072). For situational interest, direct effects were again found for TUT rate (negative), prior topic interest and value (positive), mastery achievement goals (positive), and test anxiety (negative); significant indirect effects were again found for initial topic interest and value, mind-wandering and boredom, and classroom media-multitasking habits. In general, then, the effects found in the full sample, across course domains, were representative of the effects found in only the introductory psychology classes.

## Discussion

Our study’s primary goals were: (a) to determine which academic traits, attitudes, and habits predicted undergraduates’ tendencies to report TUT experiences in class, (b) to test whether TUT rate predicted academic outcomes–course performance and situational interest–beyond the contributions of other academic individual differences, and (c) to assess whether TUT rate mediated the associations between academic individual differences and outcomes. The study’s secondary goals were to inform the (mixed) literature on whether TUT reports increase within class sessions and to extend the limited findings regarding classroom seating location and TUT rate. Specifically, we examined whether students’ TUT-report rates change systematically from the first to second half of class sessions and whether students sitting toward the front of the classroom report fewer TUTs than did those toward the back.

The study had several methodological strengths that we recommend for future studies. It used experience-sampling probes to assess *in vivo* TUTs, which demonstrate good construct validity (e.g., Kane et al., in press; Robison et al., 2019; Schubert et al., 2020). We sampled TUT reports from hundreds of students across multiple meetings of multiple courses, reflecting multiple topics, at two universities serving different populations. The design was powered to detect small correlations. Predictor constructs were assessed with multi-item measures (or multiple measures), most validated in prior research, and were assessed weeks before the outcomes to minimize reactive effects.

### Individual differences in classroom TUT rates and their mediating effects

Students reported TUTs to about 25% of probes on average, consistent with most classroom studies (e.g., Cameron & Giuntoli, 1972; Geerligs, 1995; Wammes, Boucher, et al., 2016). Individual TUT rates, however, varied widely, and were reliable across class meetings, suggesting a trait-like (or context-consistent) proclivity for reporting (if not experiencing) TUT. Multiple-group analyses that treated classrooms as groups indicated that initial interest and value in the course topic, classroom media-multitasking habits, and everyday proneness to mind-wandering and boredom predicted unique variance in probed TUT rate. TUT rate, in turn, predicted unique variance in final grades, as did classroom media-multitasking habits and test anxiety. TUT rate also predicted unique variance in students’ situational interest in the course, as did initial interest and value, mastery achievement goals, and test anxiety.

These findings replicate and extend those from Hollis and Was (2016), who found that TUT rate predicted learning from online course videos beyond the effects of topic interest, and from Kane, Smeekens, et al. (2017), who found that TUT rate predicted both learning from, and situational interest in, a laboratory video beyond the effects of initial knowledge and interest, classroom media-multitasking habits, and note-taking habits. As in other classroom studies, however, the TUT-outcome associations here were weaker than corresponding associations from the lab: TUT rate is only a modest predictor of classroom learning and situational interest.

Yet TUT rate mediated several associations between predictors and outcomes: (a) Self-reported classroom media multitasking and everyday mind-wandering and boredom proneness had significant indirect effects on course grades via TUT rate; (b) Classroom media multitasking, mind-wandering and boredom proneness, and initial topic interest and value had significant indirect effects on situational interest via TUT rate. That is, not only did TUT rate predict course outcomes beyond the contributions of numerous academic traits and habits, but some of those academic variables predicted course outcomes partially via shared variance with TUT rate.

These findings replicate and extend those of Kane, Smeekens, et al. (2017), who found in the laboratory that TUT rate mediated the indirect effects of classroom media multitasking habits on lecture learning and situational interest, and the indirect effect of initial topic interest on situational interest. In contrast, the indirect effect of initial topic interest on learning performance (i.e., course grades) via TUT rate reported by both Hollis and Was (2016) and Kane, Smeekens, et al. (2017) was in the right direction here but not significant (*p* = .085).

Classroom media-multitasking findings warrant discussion. It may not be surprising that students who multitask (i.e., engage in media use) in class are also more likely to mind-wander, perform poorly, and lack situational interest. However, Kane, Smeekens, et al. (2017) found similar associations in the laboratory, where subjects couldn’t multitask during learning. Classroom media-multitasking habits may therefore reflect a general distractibility (with distractibility causing multitasking or multitasking causing distractibility), or this distractibility may be specific to learning contexts, as media multitasking correlated here more strongly with classroom TUTs than with a general mind-wandering questionnaire.

Note, however, the potential limitation that we measured classroom media multitasking habits only by retrospective reports and not by observation or in-the-moment experience sampling. It is therefore possible that, like smartphone use (e.g., Bjerre-Nielsen et al., 2020), students misestimate the extent of their multitasking behavior in ways that influence its association with academic outcomes. We encourage further research on associations among classroom versus general media-multitasking tendencies, mind-wandering, and learning, especially research that attempts to validate classroom multitasking tendencies with observational or experience sampling data.

Finally, we note that both text anxiety and mastery goal orientation had only direct effects on study outcomes without being mediated by TUT rate. Test anxiety did not correlate significantly with TUT rate, so the lack of indirect effects on course grade or situational interest is not surprising; the literature, however, suggested a potential association between test anxiety and distracting critical thoughts that we did not find (e.g., Sarason, 1984; Zeidner, 1998). Mastery goals, in contrast, did correlate significantly with TUT rate as expected, so the lack of an indirect effect on situational interest via TUT rate likely had a different cause. Namely, mastery goals simply did not predict TUT rate in the regression models, likely due to its shared variance with other predictors, such as topic interest and value, that predicted unique variance in TUT rate. Thus, any indirect effect of mastery goals was likely obscured by collinearity with other academic predictors.

### Timecourse of TUT rates

Laboratory tasks uniformly elicit increasing TUTs with time-on-task (e.g., Kane, Smeekens, et al., 2017; Risko et al., 2012, 2013), but the time-in-class effects on TUTs in classroom studies are mixed. Most find increases (e.g., Cohen et al., 1956; Lindquist & McLean, 2011; Varao-Sousa & Kingstone, 2019), but some don’t (e.g., Wammes et al., 2019; Wammes, Boucher, et al., 2016). The present study found a small average increase from the first to second half of class meetings, consistent with most classroom studies and inconsistent with those reported by Wammes and colleagues (Wammes et al., 2019; Wammes, Boucher, et al., 2016; Wammes & Smilek, 2017). It is noteworthy that all the Wammes data come from classes taught by the same professor, who may be unusual in stemming the classroom TUT tide.

Nonetheless, the association between classroom time and TUTs may be complicated. Our study was the first to examine individual students’ TUT-report trajectories with time. Psychologists often draw conclusions from only aggregated statistics, but these estimates may poorly represent most of the contributing subjects (Grice, 2015; Grice et al., in press). As illustrated in Fig. 3, we found that the aggregate statistics were indeed obfuscating. Whereas a large minority of students showed the average increasing pattern, *more* students showed either no change or a modest decrease in TUT reports. Moreover, these individual differences might be reliable and meaningful: First-half to second-half TUT-rate change correlated weakly with course outcomes, such that students with more increasing trajectories also tended to show poorer course performance and lower situational interest. Future studies of time-on-task effects on TUTs, in classrooms and labs, should assess individual differences and whether aggregate trends sufficiently represent the trajectories of most subjects.

### Seating location and TUTs

Students sitting in the front of large classrooms tend to earn better grades than do those in the back (LaCroix & LaCroix, 2017), even in some studies that randomly assigned seats (e.g., Griffith, 1921; Perkins & Wiemann, 2005). Because sitting near the instructor may facilitate attention, we sought to add to the few, mixed findings on the association between seating location and TUTs (Lindquist & McLean, 2011; Wammes et al., 2019) by having students report their general seating location during both classroom visits. The effects were striking: TUT-report rates increased dramatically from the front third to the back third of the room (see Fig. 2).

Students chose their seats and so our findings are correlational. Seating may therefore have not affected on-task focus, but rather pre-existing differences in engagement may have influenced both students’ seating choices and TUT rates, with more engaged students sitting in front and mind-wandering less. Yet only one of our academic predictor variables varied significantly by seating location, and seating location yielded significantly different TUT rates even when statistically accounting for all predictors.

One study cannot rule out all confounds, but to the extent that we measured academic predictor constructs reasonably comprehensively, our findings limit plausible causal alternatives. Either seating location caused TUT-report variation, or an unmeasured construct acted as a third variable and caused variation in both. Intellectual ability may be among the few remaining alternatives for such a third variable, given the well-established associations between cognitive ability and TUT rate (e.g., McVay & Kane, 2012a, 2012b; Mrazek et al., 2012; Robison et al., 2020) and between cognitive ability and academic performance (e.g., Richardson et al., 2012). If future work shows seating location to influence TUTs, beyond effects of ability or engagement, it would be an efficient intervention for students with attention difficulties.

### Inferential challenges regarding the causes and consequences of TUTs

Classroom and laboratory studies of mind-wandering are inherently correlational and don’t individually allow causal conclusions, even plausible ones (e.g., lack of prior interest should elicit TUTs in class; TUTs should disrupt lecture encoding and impede learning). At the same time, because we measured TUT predictors well before TUT assessments, and because we measured TUT reports before course outcomes were determined or measured, our study design rules out some confounds.

For example, we can dismiss concerns that TUT reports or course performance reactively influenced students’ self-reported motivations, initial interests, or habits, or that performance reactively affected students’ TUT reports. Indeed, because each assessment phase was separated by weeks–in contrast to laboratory and single-session classroom studies–it is unlikely that students’ responses in any phase were artifactually influenced by a prior phase. Moreover, by statistically accounting for many plausible causes of course performance and situational interest beyond TUTs, our study modestly strengthened the evidence for the causal claim that variation in TUTs contributes to variation in learning and situational interest. Additional research must replicate these findings and account for other plausible causal constructs, such as domain knowledge (which has not fared well in classroom studies; Wammes, Seli, et al., 2016; Wammes et al., 2019; Wammes & Smilek, 2017) or cognitive ability.

Causal inference about mediation is trickier still (e.g., Bullock et al., 2010; MacKinnon, 2008; MacKinnon & Pirlott, 2015): Indirect-effects estimates are biased if not all relevant predictors and mediators are modeled. Because no study can assess all plausible predictors and mediators, our mediation findings must be considered preliminary until a larger research program supports them. Of importance, however, we reiterate the consistency of several TUT-mediation findings across the present study in ten classrooms, the Hollis and Was (2016) online-course study, and the Kane, Smeekens, et al. (2017) lab study, summarized above. These indirect effects thus appear–so far, at least–to be reasonably consistent across setting, subjects, and measurement batteries.

### Limitations and constraints on generalizability

Given the consistency of our primary findings with others across settings and populations, we expect them to generalize to adequately powered studies of undergraduate courses that are primarily lecture-based, with relatively large enrollments, with grades determined primarily by exams, and with TUTs assessed via thought probes in at least one relatively early class session. In contrast, we would be concerned about generalizing our findings to smaller interactive classrooms, to “flipped” classes that are activity-focused, and to student samples with narrower variability in interest, motivation, TUT rate, and course performance than in typical introductory courses at comprehensive universities.

Questions of generalizability seem more open with respect to course topics and culture. The present study, like many classroom-TUT studies (e.g., Lindquist & McLean, 2011; Ralph et al., 2017; Varao-Sousa & Kingstone, 2015; Wammes, Boucher, et al., 2016), investigated only psychology courses. Courses in other disciplines might evoke different TUT rates, TUT associations, or TUT-rate mediation patterns. Moreover, and consistent with enrollments in U.S. psychology courses (APA, 2020), our sample lacked gender balance, with 75% of subjects being women. We know of no studies of gender differences in TUT experiences, but TUT-rate associations with academic variables could vary with gender or other demographic variables. Similarly, most classroom TUT studies have been conducted in Western settings. Although the few studies on everyday mind-wandering in Eastern cultures suggest similar TUT experiences to those in Western cultures (Shukor, 2005; Song & Wang, 2012), successful generalization to non-Western classrooms remains an open empirical question (Henrich et al., 2010).

The most significant limitations to our study are as follows:

- As discussed above, our measure of classroom media multitasking habits relied on retrospective self-report and so reporting or memory biases may have contributed to observed associations;
- Our sample was biased toward better academic performers (i.e., average course grades for our sample, standardized against all students in the target courses, were greater than zero), perhaps because we provided only a modest participation incentive;
- Although it sampled multiple classrooms, it didn’t sample enough to afford multilevel analyses or statistical testing for differences among the classrooms or course types (i.e., introductory statistics versus introductory psychology);
- Although it sampled from two meetings per class with six to nine probes per meeting, it didn’t sample enough thoughts to allow for reliable estimates of some theoretically interesting thought-report types (e.g., lecture-related off-task thoughts; see Jing et al., 2016; Kane, Smeekens, et al., 2017);
- The study’s operational definition of mind-wandering was TUT, but there are alternative ways to define the construct that may have yielded different conclusions (Seli et al., 2018); moreover, we employed a single probe type that focused on the content of subjects’ thoughts, but probes may assess other dimensions of mind-wandering experiences (e.g., depth, intentionality, valence, dynamics) and these may sometimes elicit different results (Kane et al., in press).

Because a better understanding of off-task thought in the classroom–along with its individual-differences predictors and consequences–might lead to effective educational interventions, we encourage large-scale collaborative efforts to replicate, generalize, and extend our findings.

### Supplementary Information

ESM 1 (PDF 429 kb)

## Appendix 1

**Table:** **Number of registered, consented, and completed students in each target class, and their standardized course grades**

Target Class	Registered	Consented	Completed	Grade z-score
1 UA INTRO	396	195 (49%)	127 (32%)	0.18 (0.89)
2 UA INTRO	359	267 (74%)	217 (60%)	0.25 (0.85)
3 UA STATS	88	72 (82%)	61 (69%)	0.27 (0.72)
4 UA STATS	89	67 (75%)	62 (70%)	0.29 (0.71)
5 UB INTRO	213	98 (46%)	83 (39%)	0.38 (0.86)
6 UB INTRO	184	75 (41%)	70 (38%)	0.36 (0.86)
7 UB INTRO	162	40 (25%)	37 (23%)	0.61 (0.75)
8 UB INTRO	138	63 (46%)	59 (43%)	0.37 (0.71)
9 UB INTRO	130	65 (50%)	61 (47%)	0.25 (0.88)
10 UB STATS	133	78 (59%)	74 (56%)	0.32 (0.81)

*Note.* Registered = number of registered students in each class; Consented = number of initially consenting students; Completed = number of students completing all required components of the study (total *N* = 851); Grade z-score = mean final course grade standardized within classrooms including all registered students. Parentheses indicate either the percent (%) of registered students (columns 3 and 4) or the standard deviation of the z-score mean (column 5)

*UA* University A, *UB* University B, *INTRO* Introductory Psychology, *STATS* Psychological Statistics

## Appendix 2

**Table:** **In-class proportions of task-unrelated thoughts, for each class, averaged across both visits (total** ***N*** **= 851)**

Class Sections	n	Mean	SD	Min	Max	Skewness (SE)	Kurtosis (SE)
1 UA INTRO	127	0.31	0.22	0.00	1.00	1.02 (0.21)	1.31 (0.43)
2 UA INTRO	217	0.22	0.17	0.00	0.83	0.69 (0.17)	0.10 (0.33)
3 UA STATS	61	0.22	0.18	0.00	0.75	0.94 (0.31)	0.80 (0.60)
4 UA STATS	62	0.17	0.14	0.00	0.67	0.92 (0.30)	1.51 (0.60)
5 UB INTRO	83	0.21	0.16	0.00	0.72	0.69 (0.26)	0.56 (0.52)
6 UB INTRO	70	0.25	0.17	0.00	0.72	0.90 (0.29)	0.86 (0.57)
7 UB INTRO	37	0.31	0.17	0.00	0.78	0.65 (0.39)	0.37 (0.76)
8 UB INTRO	59	0.25	0.21	0.00	1.00	0.96 (0.31)	1.59 (0.61)
9 UB INTRO	61	0.25	0.19	0.00	0.78	0.80 (0.31)	0.21 (0.60)
10 UB STATS	74	0.22	0.17	0.00	0.89	1.50 (0.28)	3.34 (0.55)

Note. If a student attended only one visit, their proportion of task-unrelated thought for that visit was used

*UA* University A, *UB* University B, *INTRO* Introductory Psychology, *STATS* Psychological Statistics

## Footnotes

1 However, in the corresponding dissertation document, Hollis (2013) reported that interest showed a significant indirect effect on course performance via TUT rate (*p* = .03); WMC did not (*p* = .06)

2 The empirical literature on media multitasking often refers to simultaneous multitasking across multiple forms of media (e.g., Loh et al., 2016; Ophir et al., 2009). Our measure, also used in the present study, focused instead on the tendency to multitask between classroom activities and engaging in any form of media use (without specifying whether different media forms were engaged simultaneously).

3 For one 75-min class meeting, the instructor unexpectedly ended class early, allowing only five probes.

4 Items included (a) behavioral-trait measures, such as extraversion, conscientiousness, socially desirable responding, self-control, general procrastination, academic procrastination, and smartphone use, and (b) retrospective reports of behaviors (e.g., note-taking, multitasking, mind-wandering) within the studied course.

5 At a reviewer’s request, we further assessed any influence of classroom on the seating-TUT association via a 3 (seating location) × 10 (classroom) ANOVA on TUT rates for each visit. The interaction was non-significant for both visit 1, *F*(18,760) = 1.28, *p* = .193, and visit 2, *F*(18, 756) = 1.41, *p* = .117.

## References

[CR1] Acai, A. (2016). *What are residents paying attention to? An exploration of mind wandering during classroom-based teaching sessions (academic half days) in postgraduate medical education*. Unpublished Masters thesis, McMaster University (Hamilton, ON).

[CR2] Allen J, Robbins S (2010). Effects of interest-major congruence, motivation, and academic performance on timely degree attainment. Journal of Counseling Psychology.

[CR3] Allen M, Poggiali D, Whitaker K, Marshall TR, Kievit RA (2019). Raincloud plots: a multi-platform tool for robust data visualization. Wellcome Open Research.

[CR4] American Psychological Association. (2020). Degrees in Psychology [interactive data tool]. https://www.apa.org/workforce/data-tools/degrees-psychology. Accessed October 27, 2020

[CR5] Antrobus JS, Singer JL, Greenberg S (1966). Studies in the stream of consciousness: Experimental enhancement and suppression of spontaneous cognitive processes. Perceptual and Motor Skills.

[CR6] Ashcraft, M. H., & Ridley, K. S. (2005). Math anxiety and its cognitive consequences. In J. I. D. Campbell (Ed.), *Handbook of mathematical cognition* (pp. 315-327). Psychology Press.

[CR7] Beilock, S. L., Rydell, R. J., and McConnell, A. R. (2007). Stereotype threat and working memory: Mechanisms, alleviation, and spill over. *Journal of Experimental Psychology: General, 136*, 256-276. 10.1037/0096-3445.136.2.256 17500650

[CR8] Bjerre-Neilsen, A., Andersen, A., Minor, K., & Dreyer Lassen, D. (2020). The negative effect of smartphone use on academic performance may be overestimated: Evidence from a 2-year panel study. *Psychological Science, 31*(11):1351-1362. 10.1177/0956797620956613 33021885

[CR9] Bloom BS (1953). Thought-processes in lectures and discussions. Journal of General Education.

[CR10] Breed G, Colaiuta V (1974). Looking, blinking, and sitting: Nonverbal dynamics in the classroom. Journal of Communication.

[CR11] Brunson JC (2020). ggalluvial: Layered Grammar for Alluvial Plots. Journal of Open Source Software.

[CR12] Bullock JG, Green DP, Ha SE (2010). Yes, but what’s the mechanism? (Don’t expect an easy answer). Journal of Personality and Social Psychology.

[CR13] Bunce DM, Flens EA, Neiles KY (2010). How long can students pay attention in class? A study of student attention decline using clickers. Journal of Chemical Education.

[CR14] Burdett BRD, Charlton SG, Starkey NJ (2019). Mind wandering during everyday driving: An on-road study. Accident analysis and prevention.

[CR15] Cameron P, Giuntoli D (1972). Consciousness sampling in the college classroom or Is anybody listening?. Intellect.

[CR16] Casner SM, Schooler JW (2014). Thoughts in flight: Automation use and pilots’ task-related and task-unrelated thought. Human Factors.

[CR17] Cohen J, Hansel CEM, Sylvester JD (1956). Mind wandering. British Journal of Psychology.

[CR18] Conley, A. M. (2012). Patterns of motivation beliefs: Combining achievement goal and expectancy-value perspectives. *Journal of Educational Psychology, 104,* 32–47.

[CR19] Dane E (2018). Where is my mind? Theorizing mind wandering and its performance-related consequences in organizations. Academy of Management Review.

[CR20] Elliot AJ, Harackiewicz JM (1994). Goal setting, achievement orientation, and intrinsic motivation: A mediational analysis. Journal of Personality and Social Psychology.

[CR21] Elliot AJ, Church MA (1997). A hierarchical model of approach and avoidance achievement motivation. Journal of Personality and Social Psychology.

[CR22] Elliot, A. J., & Murayama, K. (2008). On the measurement of achievement goals: Critique, illustration, and application. *Journal of Educational Psychology, 100*, 613-628.

[CR23] Flake JK, Fried EI (2020). Measurement schmeasurement: Questionable measurement practices and how to avoid them. Advances in Methods and Practices in Psychological Science.

[CR24] Flake JK, Pek J, Hehman E (2017). Construct validation in social and personality research: Current practice and recommendations. Social Psychology and Personality Sciences.

[CR25] Fox, K. C. R., & Christoff, K. (2018). *The Oxford handbook of spontaneous thought: Mind-wandering, creativity, and dreaming*. Oxford University Press.

[CR26] Geerligs T (1995). Students’ thoughts during problem-based small-group discussions. Instructional Science.

[CR27] Gontier C (2017). How to prevent mind-wandering during an EVA? Presentation of a mind-wandering detection method using ECG technology in a Mars-analog environment. Acta Astronautica.

[CR28] Grice, J. W. (2015). From means and variances to persons and patterns. *Frontiers in Psychology, 6*, article 1007. 10.3389/fpsyg.2015.01007 10.3389/fpsyg.2015.01007 PMC4513286 26257672

[CR29] Grice, J. W., Medellin, E., Jones, I., Horvath, S., McDaniel, H., O’Lansen, C., & Baker, M. (in press). Persons as effect sizes. *Advances in Methods and Practices in Psychological Science*. 10.1177/25152459209229

[CR30] Griffith CR (1921). A comment upon the psychology of the audience. Psychological Monographs.

[CR31] Harackiewicz, J. M., Durik, A. M., Barron, K. E., Linnenbrink-Garcia, L., & Tauer, J. M. (2008). The role of achievement goals in the development of interest: Reciprocal relations between achievement goals, interest, and performance. *Journal of Educational Psychology, 100*, 105-122.

[CR32] Henrich J, Heine SJ, Norenzayan A (2010). The weirdest people in the world?. Behavioral and Brain Sciences.

[CR33] Hidi S (1990). Interest and its contribution as a mental resource for learning. Review of Educational Research.

[CR34] Hollis, R. B. (2013). *Mind wandering and online learning: A latent variable analysis*. Unpublished doctoral dissertation, Kent State University (Kent, OH).

[CR35] Hollis RB, Was CA (2016). Mind wandering, control failures, and social media distractions in online learning. Learning and Instruction.

[CR36] JASP Team (2020). JASP (Version 0.12.2) [Computer software].

[CR37] Jing HG, Szpunar KK, Schacter DL (2016). Interpolated testing influences focused attention and improves integration of information during a video-recorded lecture. Journal of Experimental Psychology: Applied.

[CR38] Kane MJ, Brown LE, McVay JC, Silvia PJ, Myin-Germeys I, Kwapil TR (2007). For whom the mind wanders, and when: An experience-sampling study of working memory and executive control in daily life. Psychological Science.

[CR39] Kane MJ, Gross GM, Chun CA, Smeekens BA, Meier ME, Silvia PJ, Kwapil TR (2017). For whom the mind wanders, and when, varies across laboratory and daily-life settings. Psychological Science.

[CR40] Kane, M. J., Smeekens, B. A., Meier, M., Welhaf, M., & Phillips, N. (in press). Testing the construct validity of competing measurement approaches to probed mind-wandering reports. Behavior Research Methods. 10.3758/s13428-021-01557-x PMC8613094 33835393

[CR41] Kane MJ, Smeekens BA, von Bastian CC, Lurquin JH, Carruth NP, Miyake A (2017). A combined experimental and individual-differences investigation into mind wandering during a video lecture. Journal of Experimental Psychology: General.

[CR42] LaCroix K, LaCroix S (2017). Does seat location matter? A review of the proximity effect in large and small classrooms. Community College Enterprise.

[CR43] Lindquist SI, McLean JP (2011). Daydreaming and its correlates in an educational environment. Learning and Individual Differences.

[CR44] Linnenbrink-Garcia L, Durik AM, Conley AM, Barron KE, Tauer JM, Karabenick SA, Harackiewicz JM (2010). Measuring situational interest in academic domains. Educational and Psychological Measurement.

[CR45] Locke LF, Jensen MK (1974). Thought sampling: A study of student attention through self report. Research Quarterly.

[CR46] Loh KK, Tan BZH, Lim SWH (2016). Media multitasking predicts video-recorded lecture learning performance through mind wandering tendencies. Computers in Human Behavior.

[CR47] MacKinnon, D. P. (2008). *Introduction to statistical mediation analysis*. Erlbaum.

[CR48] MacKinnon DP, Pirlott AG (2015). Statistical approaches for enhancing causal interpretation of the M to Y relation in mediation analysis. Personality and Social Psychology Review.

[CR49] McNeish DM (2018). Thanks coefficient alpha, we’ll take it from here. Psychological Methods.

[CR50] McNeish, D. M., & Kelly, K. (2019). Fixed effects models versus mixed effects models of clustered data: Reviewing the approaches, disentangling the differences, and making recommendations. *Psychological Methods, 24*, 20-35. 10.1037/met0000182 29863377

[CR51] McNeish DM, Stapleton LM (2016). The effect of small sample size on two-level model estimates: A review and illustration. Educational Psychology Review.

[CR52] McNeish DM, Stapleton LM (2016). Modeling clustered data with very few clusters. Multivariate Behavioral Research.

[CR53] McVay JC, Kane MJ (2013). Dispatching the wandering mind? Toward a laboratory method for cuing “spontaneous” off-task thought. Frontiers in Psychology.

[CR54] McVay, J. C., & Kane, M. J. (2012a). Drifting from slow to “D’oh!”: Working memory capacity and mind wandering predict extreme reaction times and executive control errors. Journal of Experimental Psychology: *Learning, Memory, and Cognition, 38*, 525-549. 10.1037/a0025896 PMC3395723 22004270

[CR55] McVay JC, Kane MJ (2012). Why does working memory capacity predict variation in reading comprehension? On the influence of mind wandering and executive attention. Journal of Experimental Psychology: General.

[CR56] Merlo KL, Wiegand KE, Shaughnessy SP, Kuykendall L, Weiss HM (2020). A qualitative study of daydreaming episodes at work. Journal of Business and Psychology.

[CR57] Muthén, L.K. and Muthén, B.O. (2012). *Mplus user’s guide* (7th ed.). Muthén & Muthén.

[CR58] Ophir E, Nass C, Wagner AD (2009). Cognitive control in media multitaskers. Proceedings of the National Academy of Sciences.

[CR59] Perkins KK, Wieman CE (2005). The surprising impact of seat location on student performance. The Physics Teacher.

[CR60] Pintrich PR, De Groot EV (1990). Motivational and self-regulated learning components of classroom academic performance. Journal of Educational Psychology.

[CR61] Pintrich, P. R., Smith, D. A. F., Garcia, T., & McKeachie, W. J. (1991). *A manual for the use of the motivated strategies for learning questionnaire (MSLQ)*. Technical Report 91-B-004, The Regents of the University of Michigan.

[CR62] Ralph BCW, Wammes JD, Barr N, Smilek D (2017). Wandering minds and wavering goals: Examining the relation between mind wandering and grit in everyday life and the classroom. Canadian Journal of Experimental Psychology.

[CR63] Revelle W, Zinbarg R (2009). Coefficients alpha, beta, omega, and the glb: Comments on Sijtsma. Psychometrika.

[CR64] Richardson M, Abraham C, Bond R (2012). Psychological correlates of university students’ academic performance: A systematic review and meta-analysis. Psychological Bulletin.

[CR65] Risko EF, Anderson N, Sarwal A, Engelhart M, Kingstone A (2012). Everyday attention: Variation in mind wandering and memory in a lecture. Applied Cognitive Psychology.

[CR66] Risko EF, Buchanan D, Medimorec S, Kingstone A (2013). Everyday attention: Mind wandering and computer use during lectures. Computers & Education.

[CR67] Robbins SB, Allen J, Casillas A, Hamme Peterson C, Le H (2006). Unraveling the differential effects of motivational and skills, social, and self-management measures from traditional predictors of college outcomes. Journal of Educational Psychology.

[CR68] Robbins SB, Lauver K, Le H, Davis D, Langley R, Carlstrom A (2004). Do psychosocial and study skill factors predict college outcomes? A meta-analysis. Psychological Bulletin.

[CR69] Robison MK, Miller AL, Unsworth N (2019). Examining the effects of probe frequency, response options, and framing within the thought-probe method. Behavior Research Methods.

[CR70] Robison, M. K., Miller, A. L., & Unsworth, N. (2020). A multi-faceted approach to understanding individual differences in mind-wandering. *Cognition, 198*. 10.1016/j.cognition.2019.104078. 10.1016/j.cognition.2019.104078 32062086

[CR71] Sanbonmatsu, D. M., Strayer, D. L., Mederois-Ward, N., & Watson, J. M. (2013). Who multi-tasks and why? Multi-tasking ability, perceived multi-tasking ability, impulsivity, and sensation seeking. *PLoS ONE, 8(1)*. 10.1371/journal.pone.0054402. 10.1371/journal.pone.0054402 PMC3553130 23372720

[CR72] Sarason IG (1984). Stress, anxiety, and cognitive interference: Reactions to tests. Journal of Personality and Social Psychology.

[CR73] Schneider M, Preckel F (2017). Variables associated with achievement in higher education: A systematic review of meta-analyses. Psychological Bulletin.

[CR74] Schoen JR (1970). Use of consciousness sampling to study teaching methods. Journal of Educational Research.

[CR75] Schubert A-L, Frischkorn GT, Rummel J (2020). The validity of online thought-probing procedure of mind wandering is not threatened by variation of probe rate and probe framing. Psychological Research.

[CR76] Seli P, Kane MJ, Smallwood J, Schacter DL, Maillet D, Schooler JW, Smilek D (2018). Mind-wandering as a natural kind: A family resemblances view. Trends in Cognitive Sciences.

[CR77] Shukor, S. (2005). *Insights into students’ thoughts during problem based learning small group discussions and traditional tutorials*. Unpublished manuscript. Retrieved March 18, 2016 from: http://www.tp.edu.sg/staticfiles/TP/files/centres/pbl/pbl_suriya_shukor.pdf

[CR78] Siegel L, Siegel LC, Capretta PJ, Jones RL, Berkowitz H (1963). Students’ thoughts during class: A criterion for educational research. Journal of Educational Psychology.

[CR79] Simmons JP, Nelson LD, Simonsohn U (2012). A 21 word solution. *Dialogue: The Official Newsletter of the*. Society for Personality and Social Psychology.

[CR80] Singer, J. L., and Antrobus, J. S. (1970). *Imaginal Processes Inventory* (revised). Center for Research in Cognition and Affect Graduate Center, City University of New York.

[CR81] Smallwood J, Fitzgerald A, Miles LK, Phillips LH (2009). Shifting moods, wandering minds: Negative moods lead the mind to wander. Emotion.

[CR82] Smallwood J, Schooler JW (2015). The science of mind wandering: Empirically navigating the stream of consciousness. Annual Review of Psychology.

[CR83] Smeekens BA, Kane MJ (2016). Working memory capacity, mind wandering, and creative cognition: An individual-differences investigation into the benefits of controlled versus spontaneous thought. Psychology of Aesthetics, Creativity, and the Arts.

[CR84] Song X, Wang X (2012). Mind wandering in Chinese daily lives – An experience sampling study. PLoS ONE.

[CR85] Stuart J, Rutherford RJD (1978). Medical student concentration during lectures. The Lancet.

[CR86] Trizano-Hermosilla I, Alvarado JM (2016). Best alternatives to Cronbach’s alpha reliability: Congeneric and asymmetrical measurements. Frontiers in Psychology.

[CR87] Varao-Sousa TL, Kingstone A (2015). Memory for lectures: How lecture format impacts the learning experience. PLoS ONE.

[CR88] Varao-Sousa, T. L., & Kingstone, A. (2019). Are mind wandering rates an artifact of the probe-caught method? Using self-caught mind wandering in the classroom to test, and reject, this possibility. *Behavior Research Methods, 51*, 235-242. 10.3758/s13428-018-1073-0 29946951

[CR89] Walker, H. E. K., & Trick, L. M. (2018). Mind-wandering while driving: The impact of fatigue, task length, and sustained attention abilities. *Transportation Research Part F, 59*, 81-97.

[CR90] Wammes JD, Boucher PO, Seli P, Cheyne JA, Smilek D (2016). Mind wandering during lectures I: Changes in rates across an entire semester. *Scholarship of Teaching and Learning in*. Psychology.

[CR91] Wammes JD, Ralph BCW, Mills C, Bosch N, Duncan TL, Smilek D (2019). Disengagement during lectures: Media multitasking and mind wandering in university classrooms. Computers & Education.

[CR92] Wammes JD, Seli P, Cheyne JA, Boucher PO, Smilek D (2016). Mind wandering during lectures II: Relation to academic performance. Scholarship of Teaching and Learning in Psychology.

[CR93] Wammes JD, Smilek D (2017). Examining the influence of lecture format on degree of mind wandering. Journal of Applied Research in Memory and Cognition.

[CR94] Was CA, Hollis RB, Dunlosky J (2019). Do students understand the detrimental effects of mind wandering during online learning?. Computers & Education.

[CR95] Zeidner, M. (1998). *Test anxiety: The state of the art*. Plenum Press.

