# Supplementary material for: Individual differences in task-unrelated thought in university classrooms
Source: Mem Cognit. 2021 Apr 22;49(6):1247–66. doi: 10.3758/s13421-021-01156-3 (PMC8313470; doi:10.3758/s13421-021-01156-3)
Supplement: Supplementary file 1 — (PDF 429 kb) [file 13421_2021_1156_MOESM1_ESM.pdf]

SUPPLEMENTAL INFORMATION

SUPPLEMENTAL TABLE S1

In each class section, descriptive statistics for in-class proportion of task-unrelated thoughts, separately for the first and second classroom visits

Class Visits	Class Sections	n	Mean	SD	Min	Max	Skewness (SE)	Kurtosis (SE)
First Visit								
	1 UA INTRO	113	0.30	0.22	0.00	1.00	0.86 (0.23)	1.26 (0.45)
	2 UA INTRO	201	0.20	0.17	0.00	0.67	0.38 (0.17)	-0.70 (0.34)
	3 UA STATS	57	0.26	0.21	0.00	0.88	0.71 (0.32)	0.29 (0.62)
	4 UA STATS	58	0.18	0.15	0.00	0.67	0.86 (0.31)	0.86 (0.62)
	5 UB INTRO	81	0.20	0.17	0.00	0.67	0.39 (0.27)	-0.60 (0.53)
	6 UB INTRO	63	0.24	0.19	0.00	0.78	0.85 (0.30)	0.55 (0.59)
	7 UB INTRO	33	0.32	0.19	0.00	0.78	0.15 (0.41)	-0.14 (0.80)
	8 UB INTRO	55	0.22	0.19	0.00	0.67	0.57 (0.32)	-0.64 (0.63)
	9 UB INTRO	59	0.24	0.20	0.00	0.78	0.79 (0.31)	0.05 (0.61)
	10 UB STATS	71	0.21	0.21	0.00	1.00	1.32 (0.28)	2.60 (0.56)

(Supplemental Table S1 continues below)

SUPPLEMENTAL TABLE S1. (continued)

Class Visits	Class Sections	n	Mean	SD	Min	Max	Skewness (SE)	Kurtosis (SE)
Second Visit								
	1 UA INTRO	113	0.29	0.25	0.00	1.00	0.95 (0.23)	0.51 (0.45)
	2 UA INTRO	208	0.24	0.21	0.00	0.83	0.68 (0.17)	-0.23 (0.34)
	3 UA STATS	59	0.20	0.21	0.00	0.83	1.43 (0.31)	2.22 (0.61)
	4 UA STATS	55	0.16	0.16	0.00	0.67	0.92 (0.32)	0.70 (0.63)
	5 UB INTRO	77	0.21	0.18	0.00	1.00	1.36 (0.27)	4.10 (0.54)
	6 UB INTRO	66	0.27	0.21	0.00	0.89	0.87 (0.30)	0.62 (0.58)
	7 UB INTRO	34	0.29	0.21	0.00	1.00	1.11 (0.40)	2.67 (0.79)
	8 UB INTRO	50	0.27	0.25	0.00	1.00	1.01 (0.34)	0.66 (0.66)
	9 UB INTRO	58	0.27	0.23	0.00	0.78	0.65 (0.31)	-0.56 (0.62)
	10 UB STATS	72	0.23	0.21	0.00	0.78	0.86 (0.28)	0.14 (0.56)

SUPPLEMENTAL TABLE S2

Mean (standard deviation in parentheses) scores and ANOVA results for each academic predictor measure for students seated in the front, middle, versus back third of classroom rows, for each of the two probed class meetings.

	Seating in First Probed Class Meeting			
Outcome	Front Third	Middle Third	Back Third	ANOVA Results
	(n = 274)	(n = 248)	(n = 268)	df(2,787)
Note Taking Skill	3.55 (0.50)	3.46 (0.46)	3.49 (0.48)	F= 2.02, p=.133
Classroom Media Multitasking	1.88 (0.75)	2.06 (0.86)	2.83 (0.85)	**F=17.45, p<.001**
Topic Interest and Value	3.85 (0.69)	3.82 (0.67)	3.75 (0.71)	F= 1.50, p=.223
Mastery Goals	3.97 (0.65)	3.92 (0.65)	3.84 (0.63)	F= 2.78, p=.063
Course Self-Efficacy	3.89 (0.53)	3.85 (0.53)	3.86 (0.54)	F= 0.55, p=.579
Test Anxiety	3.29 (1.09)	3.35 (1.05)	3.44 (1.07)	F= 1.35, p=.260
Mind-Wandering & Boredom	3.04 (0.47)	3.08 (0.46)	3.12 (0.46)	F= 1.81, p=.165
Multitasking Beliefs	2.88 (0.48)	2.84 (0.42)	2.93 (0.46)	F= 2.47, p=.085
Performance Goals	4.07 (0.80)	4.08 (0.73)	4.08 (0.72)	F= 0.04, p=.963

(Supplemental Table S2 continues below)

SUPPLEMENTAL TABLE S2 (continued)

	Seating in Second Probed Class Meeting			
Outcome	Front Third	Middle Third	Back Third	ANOVA Results
	(n = 271)	(n = 240)	(n = 275)	df(2,783)
Note Taking Skill	3.53 (0.50)	3.45 (0.46)	3.52 (0.44)	$F = 1.63, p = .196$
Classroom Media Multitasking	1.87 (0.75)	2.14 (0.85)	2.25 (0.84)	**$F = 15.59, p < .001$**
Topic Interest and Value	3.82 (0.70)	3.79 (0.72)	3.79 (0.66)	$F = 0.21, p = .814$
Mastery Goals	3.92 (0.62)	3.93 (0.64)	3.88 (0.65)	$F = 0.47, p = .625$
Course Self-Efficacy	3.90 (0.53)	3.83 (0.53)	3.88 (0.53)	$F = 1.01, p = .364$
Test Anxiety	3.23 (1.12)	3.39 (1.04)	3.40 (1.04)	$F = 2.28, p = .103$
Mind-Wandering & Boredom	3.04 (0.45)	3.10 (0.46)	3.09 (0.45)	$F = 1.35, p = .261$
Multitasking Beliefs	2.86 (0.48)	2.84 (0.44)	2.93 (0.43)	**$F = 3.34, p = .036$**
Performance Goals	4.11 (0.75)	4.03 (0.80)	4.07 (0.70)	$F = 0.77, p = .462$

Note: Statistically significant results are presented in bolded type. df = degrees of freedom for analysis of variance (ANOVA).

SUPPLEMENTAL TABLE S3

Multiple group analysis results (with groups corresponding to only the 7 sampled introductory psychology classrooms; total N = 654) for the three mediating or outcome variables: classroom TUT rate, final course grade, and end-of-semester situational interest.

	Classroom TUT rate			Final Course Grade			Situational Interest		
Predictor	B	SE	*p*	B	SE	*p*	B	SE	*p*
Note-Taking Skill	−.029	.017	.086	.145	.084	.083	.093	.069	.180
Classroom Media Multitasking	**.063**	**.009**	**<.001**	**−.127**	**.042**	**.002**	−.010	.031	.751
Topic Interest and Value	**−.042**	**.014**	**.002**	−.114	.061	.061	**.530**	**.051**	**<.001**
Achievement Goals, Mastery	.002	.011	.888	**.120**	**.056**	**.032**	**.099**	**.041**	**.016**
Course Self-Efficacy	.025	.015	.100	.105	.073	.148	−.046	.059	.434
Test Anxiety	.001	.008	.894	**−.138**	**.034**	**<.001**	**−.065**	**.025**	**.008**
Mind-Wandering & Boredom	**.058**	**.017**	**<.001**	.107	.082	.190	.093	.061	.127
Classroom TUT Rate				**−.443**	**.189**	**.020**	**−.911**	**.157**	**<.001**

Note: Statistically significant coefficients are presented in bolded type. TUT = task-unrelated thought. B = unstandardized coefficient estimate; SE = standard error.

SUPPLEMENTAL TABLE S4

Multiple group analysis results (with groups corresponding to only the 7 sampled introductory psychology classrooms; total N = 654) for testing the indirect effects for each predictor variable on each of the two outcome variables—final course grade and end-of-semester situational interest—with classroom TUT rate as the mediator variable.

	Final Course Grade			Situational Interest		
Predictor	B	SE	*p*	B	SE	*p*
Note-Taking Skill	.013	.009	.154	.026	.016	.098
Classroom Media Multitasking	**−.028**	**.012**	**.023**	**−.057**	**.012**	**<.001**
Topic Interest and Value	.019	.010	.068	**.039**	**.014**	**.005**
Achievement Goals, Mastery	−.001	.005	.888	−.001	.010	.888
Course Self-Efficacy	−.011	.008	.186	−.023	.014	.109
Test Anxiety	.000	.003	.894	−.001	.007	.894
Mind-Wandering & Boredom	−.026	.014	.072	**−.053**	**.019**	**.004**

Note: Statistically significant coefficients are presented in bolded type. TUT = task-unrelated thought. B = unstandardized coefficient estimate; SE = standard error.
